# Antigen Spreading via Localized Administration Enhances Adoptive TCR‐T Cell Therapy in Pancreatic Cancer

**DOI:** 10.1002/advs.76135

**Published:** 2026-06-19

**Authors:** Junming Huang, Qin Wang, Zhuo Yao, Qing Wang, Yongtao Ji, Minqi Yang, Meng Wang, Shiyi Shao, Xinyu Zhao, Fu Zhang, Tingbo Liang, Qida Hu

**Affiliations:** ^1^ Department of Hepatobiliary and Pancreatic Surgery School of Medicine the First Affiliated Hospital Zhejiang University Hangzhou Zhejiang China; ^2^ Zhejiang Provincial Key Laboratory of Pancreatic Disease School of Medicine the First Affiliated Hospital Zhejiang University Hangzhou Zhejiang China; ^3^ Department of Breast Surgery the First Affiliated Hospital School of Medicine Zhejiang University Hangzhou Zhejiang China; ^4^ The Innovation Center For the Study of Pancreatic Diseases Hangzhou China; ^5^ Zhejiang University Cancer Center Zhejiang University Hangzhou China; ^6^ MOE Joint International Research Laboratory of Pancreatic Diseases Hangzhou China

**Keywords:** antigen presentation, antigen, cancer, cancer research, cd8, cell therapy, immune system, medicine, pancreatic cancer

## Abstract

Cancer vaccines have demonstrated initial effectiveness in the treatment of human cancers, still the low mutational burden, which leads to a deficiency of well‐characterized antigens expressed within tumors capable of mediating tumor rejection, poses a significant challenge in pancreatic cancer. Here, we introduce an ionic liquid ILvax for localized tumor administration, which elicits a systemic and antigen‐specific anti‐tumor immune response, thereby generating a vaccine‐like effect. Type 1 conventional dendritic cells (cDC1) is the determinant for systemic immune response. ILvax ablation facilitates migration of tumor antigen‐carrying cDC1 to peripheral lymphoid organs, thus initiating the cDC1‐mediated antigen presentation cascade that results in an amplified antigen‐specific T cells response. Meanwhile, cDC1‐mediated antigen spreading induced by ILvax enabled the expansion of adoptive TCR‐T while shifting TCR‐T metabolism toward oxidative phosphorylation (OXPHOS). Localized tumor administration with ILvax simultaneously enhances the functionality of both endogenous CD8^+^ T cells and adoptive TCR‐T cells, offering a clinically translatable collaborative strategy against pancreatic cancer.

## Graphical abstract

Cancer vaccines face limitations in pancreatic cancer due to insufficient tumor antigens. This ionic liquid ILvax via local tumor delivery triggers robust cDC1‐dependent systemic anti‐tumor immunity. It boosts endogenous CD8^+^ T cells and optimizes adoptive TCR‐T function, providing a promising synergistic therapeutic strategy.

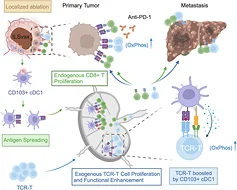

## 1 Introduction

Cancer immunotherapy leverages the patient's immune system to target and combat tumors effectively. Despite significant advancements in the effective application of immunotherapy for the treatment of various solid tumors, its utilization in addressing pancreatic cancer remains challenging [1, 2, 3]. A successful cancer immunotherapy requires the activation of the cancer‐immunity cycle (CIC) [4, 5]. This cycle comprises the release of tumor antigens from cancer cells, antigen capture and processing by antigen‐presenting cells (APCs), the generation of antigen‐specific T cells, and finally, the lysis of tumor cell by antigen‐specific T cells. In situ cancer vaccination represents a class of immunotherapeutic interventions that harness endogenous tumor‐associated antigens (TAAs) localized within native tumor niches to orchestrate de novo systemic immunity while establishing immunological memory, thereby reconstituting the defective cancer‐immunity cycle through sequential phase transitions [6, 7, 8]. Antigen spreading refers to the induction and amplification of immune responses directed against secondary antigens that are distinct from the original therapeutic target, representing the initial phase in the cancer‐immunity cycle and the key to in situ cancer vaccination [9, 10].

Locoregional therapy initiates the cancer‐immunity cycle by enhancing antigen spreading, mimicking the process of in situ vaccination [11, 12]. This mechanism improves processing and presentation of antigens mediated by varieties of antigen presentation cells (APCs), including cDC1 as a predominant APC subpopulation, which further generates amplified antigen‐specific T cell responses [13, 14]. In our previous work, we have explored the possibility using ionic liquid (IL) as a novel ablative agent to achieve immune responses and antigen spreading [15]. Tumor‐derived antigens after tumor cell lysis following IL administration contribute to TAA enrichment, which correlates to the induction of vaccination‐like responses [15]. Therefore, IL administration could be applied to the solid tumors to induce a vaccination effect, and intratumoral injection of IL could be defined as in situ vaccine (ILvax). In the setting of this work, the process of antigen spreading is currently being enhanced post ILvax ablation, and CD103^+^ cDC1 serves as a crucial determinant in the restoration of the cancer‐ immunity cycle, facilitating the transformation of tumor antigens into peripheral lymphoid organs via cDC1 and promoting the expansion of antigen‐specific T cells. Preliminary summary, employing strategies to locally ablate tumors using ILvax while simultaneously eliciting endogenous T cell responses against tumor‐specific antigens presents a promising approach to overcome pancreatic cancer with low mutational burden.

The remarkable success of adoptive cell transfer (ACT) cell therapy in certain hematologic malignancies has prompted investigations into adoptive cell transfer for patients with refractory solid tumors [16]. However, the limited expansion and persistence of CAR‐T and TCR‐T cells in vivo, combined with intrinsic characteristics of solid tumors, such as antigenic heterogeneity and immunosuppressive microenvironment, impede the efficacy of antigen‐targeted treatments [17, 18, 19]. Vaccines aim to activate the immune system against cancer cells, showing potential in conjunction with adoptive T cell therapy for solid tumor treatments [20]. For example, CAR antigen vaccine promotes efficient in vivo expansion, superior functionality, and memory formation of CAR‐T cells in pre‐clinical and clinical stages [21, 22]. In addition, Localized tumor‐directed interventions with intrinsic in situ vaccination properties, including oncolytic virus, physical ablation, and chemical ablation therapy facilitates the clonal expansion and metabolic reprogramming of CAR‐T cells in vivo, thereby augmenting their therapeutic efficacy against solid tumors [23, 24, 25]. Although numerous localized tumor administrations exist that enhance adoptive T cells therapy, the underlying mechanism remains poorly understood.

It was clarified that the systemic anti‐tumor responses induced by localized tumor administration with ILvax, which are analogous to those of vaccines, stem from the enhancement of the initiation step in the cancer‐immunity cycle, specifically antigen spreading. Meanwhile, it is noteworthy that cDC1‐mediated antigen spreading post ILvax ablation can both boost endogenous CD8^+^ T cells and adoptive TCR‐T cells functionality, synergistically inhibiting the progression of primary tumors as well as metastatic tumors. The observation that localized tumor administration enhances the activation and expansion of adoptive TCR‐T cells carries substantial significance for advancing the application of adoptive T‐cell therapy in pancreatic cancer.

## 2 Results

### 2.1 ILvax Synthesis

ILvax was synthesized through salt metathesis utilizing a 1:1 molar ratio of choline bicarbonate and geranic acid, creating the ionic liquid as previously documented (Figure S1A) [15, 26]. ^1^H NMR characterization was performed and was found to be consistent with the previously published spectra (Figure S1B).

#### 2.1.1 ILvax Ablation Of Pancreatic Cancer Modulates Intratumoral Immune Microenvironment

**FIGURE 1:**
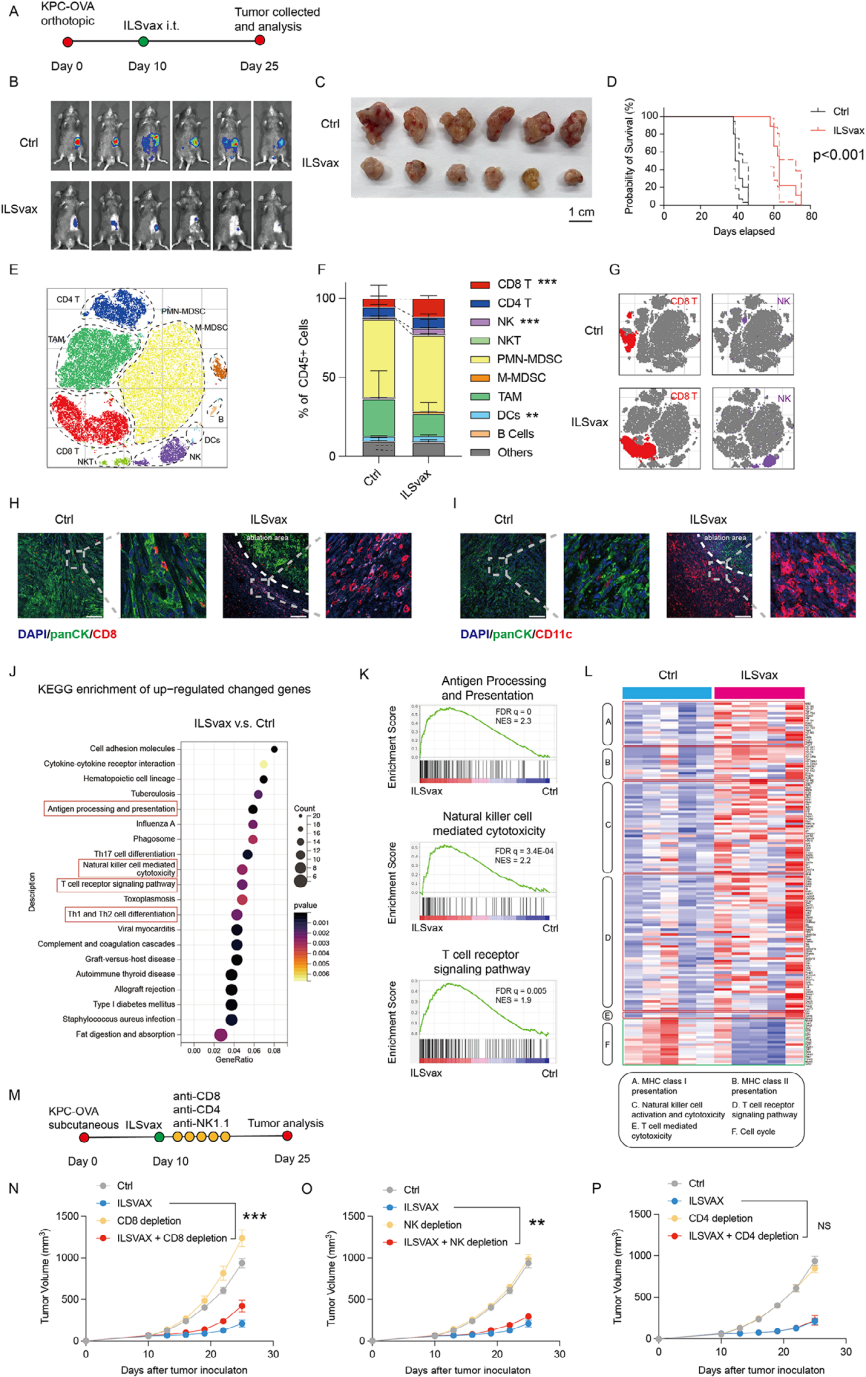
ILvax ablation of pancreatic cancer modulates intratumoral immune microenvironment. (A) Illustration of therapy in orthotopic KPC tumor‐bearing C57BL/6 mice. (B) In vivo bioluminescence imaging the orthotopic tumors in mice after the indicated treatment on day 25, n = 6. (C) The image of harvested tumors from mice after indicated administration. (D) The Kaplan‐Meier survival curves of mice after the indicated treatment, which were obtained in another parallel experiment, n = 10, log‐rank (Mantel‐Cox) test, *p* < 0.001 ILvax vs. Ctrl. (E) A t‐distributed stochastic neighbor embedding (tSNE) analysis showing the intratumoral immune cell clusters. (F) Quantitative analysis of intratumoral immune cells in different treatment groups, n = 6, two‐tailed student's *t*‐test, ^***^ *p* < 0.001, ^**^ *p* < 0.01, ILvax vs. Ctrl. (G) Representative images showing the t‐SNE analysis of CD8 and NK1.1 markers in tumor across different treatment groups. (H and I) Representative images showing the immunofluorescence multicolour‐labelled CD8, CD11c and panCK‐positive cells in pancreatic tumor tissue after the indicated treatment. (J and K) the KEGG‐GSEA enrichment of DEGs between the PBS‐ and ILvax‐ treated tumors. NES, normalized enrichment score. (L) Heatmap of differentially expressed genes (DEGs) between PBS‐ and ILvax‐treated tumors via transcriptome analysis. (M) Illustration of therapy in orthotopic KPC tumor‐bearing C57BL/6 mice. (N, O and P) The antitumor response induced by ILvax depends on CD8^+^ T cells and NK cells partially, n = 6, Data are presented as mean ± SD, *p* values were determined by two‐way ANOVA with Tukey's post–hoc test, ^***^ *p* < 0.001, ^**^ *p* < 0.01, ^*^ *p* < 0.05. NS, no significance. Source data are provided as a Source Data file.

To investigate the potential therapeutic effects of ILvax, we conduct ultrasound‐guided local injections of ILvax into orthotopic pancreatic cancer in immunocompetent mice (Figure 1A), followed by bioluminescence imaging. Expectedly, ILvax markedly impedes the advancement of orthotopic pancreatic cancer (Figure 1B, C and Figure S1C) and improves the overall survival of mice (Figure 1D).

We further explored the effect of ILvax ablation on the tumor microenviroment (TME) via spectral flow cytometry. The t‐SNE analysis, followed by the quantification of immune cells, reveals that ILvax stimulates the tumor microenvironment, resulting in an increased proportion of CD8^+^ T cells, NK cells, and DCs among the total immune cell population (Figure S1D–G). Immunofluorescence staining of the samples demonstrated that CD8^+^ T cells and DCs accumulated mainly at the boundary between the ablation zone and the residual tumor area (Figure 1H, I). RNA‐seq data revealed that ILvax ablation significantly altered the orthotopic pancreatic cancer transcriptome profile. Kyoto Encyclopedia of Genes and Genome (KEGG) and Gene set enrichment analysis (GSEA) indicated that antigen process and presentation, IFN responses, natural killer cell mediated cytotoxicity and T cell receptor signaling pathway were significantly enhanced after ILvax localized ablation therapy (Figure 1J, K and Figure S1F). In addition, the expression levels of various immunoregulatory genes, including B2m, CD74 and Ctss in antigen process and presentation, Nkg7 in natural killer cell mediated cytotoxicity, CD3e, CD8, CD28, Prf1 and Gzmb in T cell receptor signaling and cytotoxicity were dramatically elevated (Figure 1L).

To identify the immune phenotype induced ILvax whether has an impact on oncological management, we systematically depleted CD8^+^ T cells, CD4^+^ T cells, or NK cells population before treatment (Figure 1M). Flow cytometry is employed to confirm the elimination of specific target cells within tumors (Figure S1G). Compared with IgG treatment, CD4^+^ T cells depletion did not alter the inhibitory effect of the ILvax therapy, NK cells depletion had a moderate impact on tumor growth following ILvax treatment and CD8 T cells depletion significantly impacts the tumor growth (Figure 1N, O, P, Figure S1H, I).

ILvax exerts its antitumor effects through a dual mechanism involving localized tumor ablation and the alleviation of immunosuppressive phenotype.

#### 2.1.2 ILvax Ablation Of Primary Tumor Elicits Vaccine‐Like Systemic Effects

**FIGURE 2:**
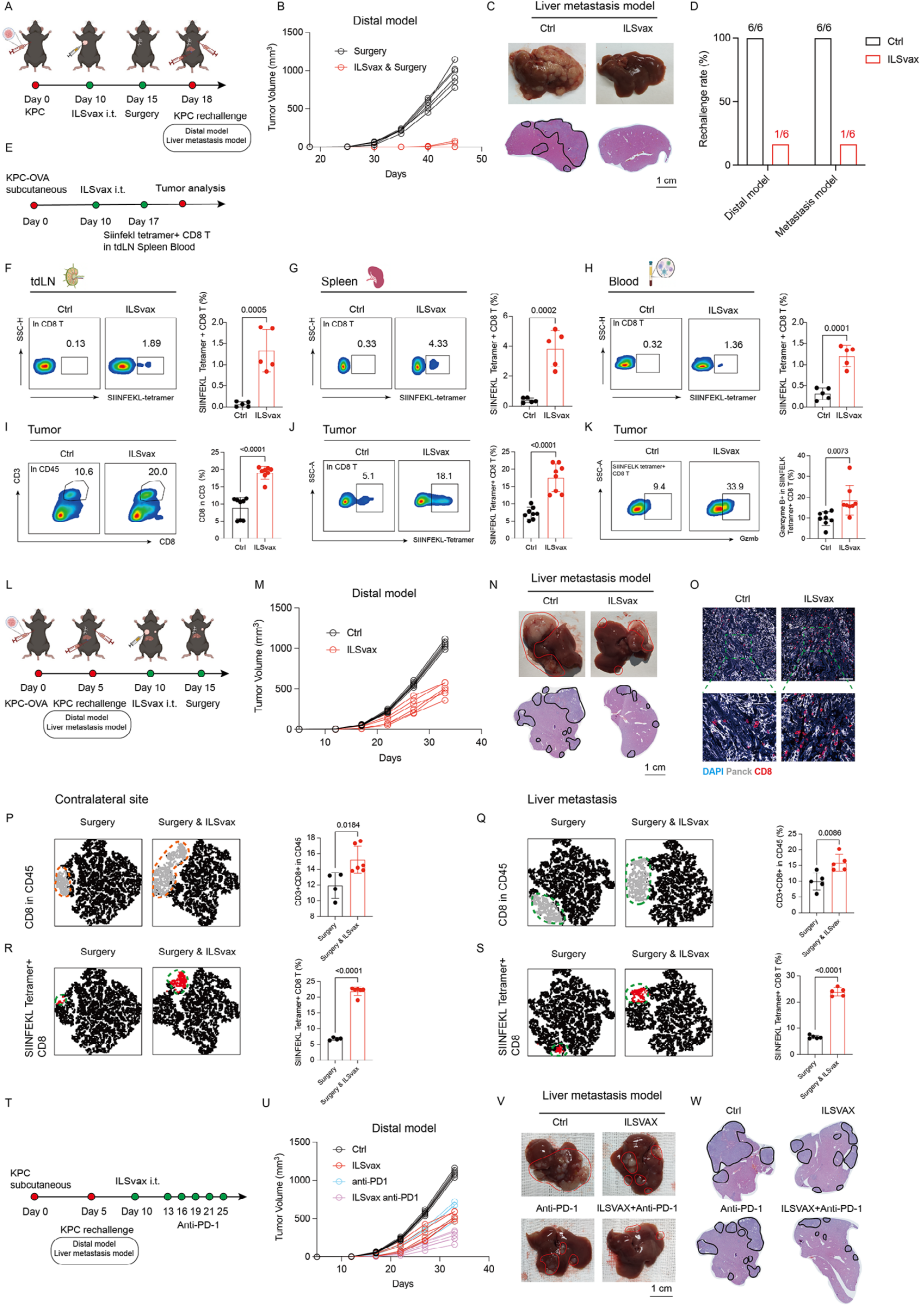
ILvax ablation of primary tumor elicits vaccine‐like systemic effects. (A) Illustration of post‐surgery rechallenge modal in KPC tumor‐bearing C57BL/6 mice. (B) Individual tumor growth curves in different groups. (C) Representative images and HE staining of liver metastasis tumors. (D) Rechallenge rate of distal and metastasis model. (E) Illustration of therapy in subcutaneous KPC‐OVA tumor‐bearing C57BL/6 mice. (F, G and H) Representative flow cytometry plots and quantitative analysis of SIINFEKL tetramer^+^ CD8^+^ T cells in draining lymph nodes (dtLN), spleens and peripheral blood. (I) Representative flow cytometry plots and quantitative analysis of Cytotoxic T lymphocytes (CTLs) in tumors in different groups (gated on CD45^+^ cells). (J) Representative flow cytometry plots and quantitative analysis of SIINFEKL tetramer^+^ CD8^+^ T cells in tumors in different groups (gated on CD45^+^CD3^+^CD8^+^ cells). (K) Representative flow cytometry plots and quantitative analysis of granzyme B^+^ CD8^+^ T cells in tumors in different groups (gated on CD45^+^CD3^+^CD8^+^ cells). (L) Illustration of pre‐surgery rechallenge model in KPC tumor‐bearing C57BL/6 mice. (M) Individual tumor growth curves in different groups. (N) Representative images and HE staining of liver metastasis tumors in different groups. (O) Representative images showing the immunofluorescence multicolour‐labelled CD8 and panCK‐positive cells in live metastasis tumor tissues after the indicated treatment. (P and Q) Representative t‐SNE plots and quantitative analysis of CD8 markers in contralateral tumors and liver metastasis tumors across different treatment groups. (R and S) Representative t‐SNE plots and quantitative analysis of SIINFEKL tetramer markers in contralateral tumors and liver metastasis tumors across different treatment groups. (T) Illustration of combination therapy in subcutaneous KPC tumor‐bearing C57BL/6 mice. (U) Individual tumor growth curves in different groups. (V and W) Representative images and HE staining of liver metastasis tumors in different treatment groups. Data are presented as mean ± SD (F‐K, and P‐S) and analysed by two‐tailed unpaired student's *t*‐test. Source data are provided as a Source Data file.

The localized tumor administration by ILvax effectively reactivated the cancer immune cycle, thereby inducing an abscopal effect, characterized by shrinkage of untreated tumors occurs concurrently with shrinkage of tumors within the area of the localized administration. We verified the existence of the abscopal effect by constructing two tumor rechallenge models, including distal model and liver metastasis modal (Figure 2A). Briefly, ILvax was used to treat the KPC mice with subcutaneous tumors on the right side. Three days later, the primary tumors were removed, and then distant metastasis models and liver metastasis models were established. Unexpectedly, ILvax treatment of the primary tumor significantly reduced the incidence of tumors in the left side and liver metastasis (Figure 2B, C, Figure S2A, B). Compared with 6/6 in the distal model and 6/6 in the liver metastasis model in the control group, the incidence of rechallenge tumors was reduced to 1/6 in the distant model and 1/6 in the liver metastasis model in treatment group (Figure 2D and Figure S2C).

The manifestation of the abscopal effect signifies the activation of the cancer immune cycle. Upon examination of peripheral lymphoid organ, the spleens, we observed that treatment with ILvax for the primary tumor resulted in an increased generation of both T cells with effector memory phenotype (Tem) and T cells with central memory phenotype (Tcm) (Figure S2D–F). The effective induction of antigen‐specific T cell generation subsequent to antigen spreading is crucial for the tumor immune cycle. To observe antigen spreading and antigen‐specific T cells generation after ILvax ablation, we constructed KPC cells expressing a model antigen Ovalbumin (OVA 256–274), KPC‐OVA. 7 days after ILvax localizd administration of subcutaneous KPC‐OVA tumors in immunocompetent mice, we detected the expansion of antigen‐specific CD8^+^ T cells in peripheral lymphoid organs, including tumor‐draining lymph nodes and spleen, and peripheral blood (Figure 2E). SIINFEKL tetramer^+^ CD8^+^ T cells were scarcely detectable in the absence of any tumor treatment. However, following ILvax treatment of the primary KPC‐OVA tumor, we observed a significant increase of SIINFEKL tetramer^+^ CD8^+^ T cells within peripheral lymphoid organs and peripheral blood (Figure 2F–H). In addition, we found that ILvax localized ablation of tumors alleviated the intratumoral immunosuppressive microenvironment, which triggered the expansion of antigen‐specific T cells and the enhancement of their cytotoxicity (Figure 2I–K).

To further elucidate the immune impact of primary tumor localized administration on the contralateral tumors and liver metastasis tumors, we developed a novel tumor rechallenge model, advancing the construction of contralateral and liver metastasis models prior to ILvax treatment for primary tumors (Figure 2L). It was anticipated that ILvax administration of primary tumors effectively inhibited the progression of both contralateral and liver metastatic tumors (Figure 2M, N, Figure S2G–I). Immunofluorescence staining of liver metastatic tumors revealed an increase in CD8^+^ T cells infiltration (Figure 2O). Spectral flow cytometry analysis demonstrated that the proportion of CD8^+^ T cells within the total immune cell population (CD45^+^ cells) increased in both contralateral tumors and liver metastatic tumors. Specifically, in the contralateral tumor, the percentage of CD8^+^ T cells was approximately 12% in the control group and 15% in the treatment group (Figure 2P). Similarly, for liver metastatic tumors, it was around 10% in the control group and 15% in the treatment group (Figure 2Q). In addition, the proportion of SIINFEKL tetramer^+^ CD8^+^ T cells within total CD8^+^ T cells increased in both contralateral tumors and liver metastatic tumors. Specifically, in the contralateral tumor, the percentage of SIINFEKL tetramer^+^ CD8^+^ T cells was approximately 6% in the control group and 20% in the treatment group (Figure 2R). Similarly, for liver metastatic tumors, it was around 8% in the control group and 22% in the treatment group (Figure 2S).

The expansion of antigen‐specific T cells induced by ILvax through antigen spreading may be responsible for the inhibition of contralateral tumor progression as well as liver metastatic tumors.

ILvax, when used in conjunction with anti‐PD‐1 immunotherapy, significantly enhanced the therapeutic efficacy against primary tumors. In comparison to monotherapy, immunohistochemical analyses demonstrated that combination therapy more effectively inhibited tumor proliferation, promotes CD8^+^ T cells infiltration and increased their cytotoxic activity for tumor cells (Figure S2M–O). Meanwhile, the augmentation of antigen‐specific CD8^+^ T cells infiltration via cancer immune circle improved the efficacy of anti‐PD‐1 immunotherapy in both contralateral tumors (Figure 2U, Figure S2J, K) and liver metastases (Figure 2V, W and Figure S2L).

ILvax ablation of primary tumors successfully elicits vaccine‐like systemic effects, thereby promoting the expansion of antigen‐specific CD8^+^ T cells and further enhancing systemic anti‐tumor responses.

#### 2.1.3 Identification of cDC1 is Indispensable For Systemic Anti‐Tumor Responses of ILvax Ablation

**FIGURE 3:**
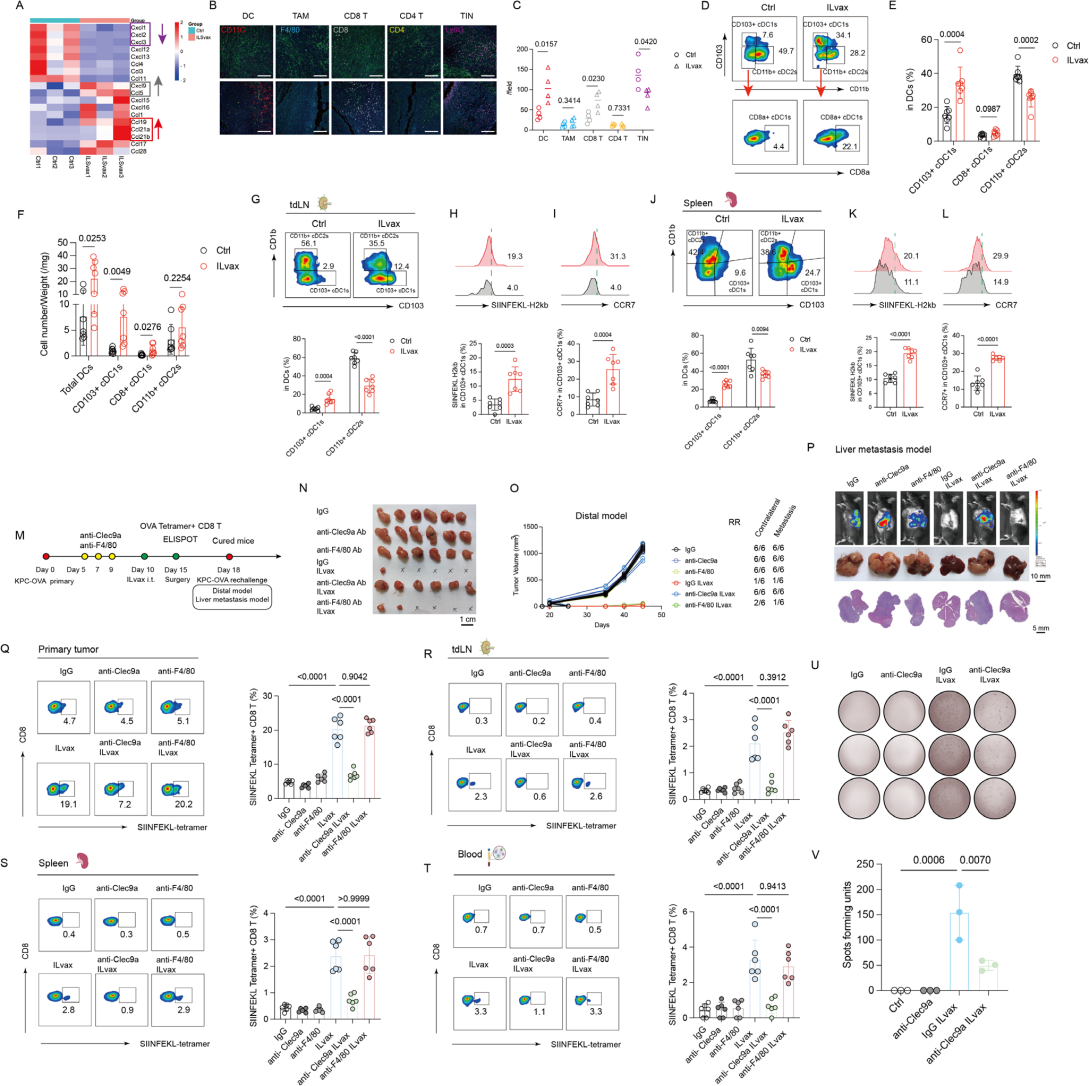
Identification of cDC1 is indispensable for systemic anti‐tumor responses of ILvax ablation. (A) Heatmap of chemokines expression of tumor after the indicated treatment via transcriptome analysis. (B) Representative images and (C) quantitative analysis showing the immunofluorescence multicolour‐labelled CD11c, F4/80, CD8, CD4 and Ly6G‐positive cells in pancreatic tumor tissues within 2 days after the indicated treatment. (D) Representative flow cytometry plots and (E) quantitative analysis of CD103^+^ cDC1, CD8a^+^ cDC1 and CD11b^+^ cDC2 in tumors in different groups (gated on CD45^+^ CD11c^+^ MHC II^+^ cells). (F) Absolute count of total DCs, CD103^+^ cDC1, CD8a^+^ cDC1 and CD11b^+^ cDC2 in tumor tissues within 2 days after the indicated treatment. (G and J) Representative flow cytometry plots and quantitative analysis of CD103^+^ cDC1 and CD11b^+^ cDC2 in dtLN and spleen after the indicated treatment (gated on CD45^+^ CD11c^+^ MHC II^+^ cells). (H, I, K and L) Representative histogram and quantitative analysis of SIINFEKL‐H2kb and CCR7 expression in CD103^+^ cDC1. (M) Illustration of therapy in KPC‐OVA tumor‐bearing C57BL/6 mice. (N) The photograph and individual tumor growth curves of harvested tumors from distal model at the end of treatment course on day 46. (P) Representative bioluminescence images, photograph and H&E staning of liver metastasis tumors. (Q, R, S and T) Representative flow cytometry plots and quantitative analysis of SIINFEKL tetramer^+^ CD8^+^ T cells in tumors in different groups (gated on CD45^+^ CD3^+^ CD8^+^ cells). (U and V) Images of IFN‐γ spot and quantitative analysis of spots counting, n = 3. Data are presented as mean ± SD, p value were determined by two‐tailed unpaired student's t‐test (C, E, F, H, I, K, and L) and one‐way ANOVA with Tukey's multiple comparisons test (Q, R, S, T, and V). Source data are provided as a Source Data file. [Correction added on 14 July 2026, after first online publication: Figure 3 was update in this version.]

To elucidate the mechanism by which ILvax activates systemic anti‐tumor responses, we performed transcriptome sequencing on primary tumors after ILvax ablation treatment. Through the screening of numerous chemokines, we observed a downregulation in the expression of Cxcl1, Cxcl2, and Cxcl3, which are known to attract myeloid‐derived suppressor cells (MDSCs). Conversely, there was an upregulation in the expression of Cxcl9 and Ccl5 that attract CD8 T cells, as well as an increase in the expression of Ccl19 and Ccl21 that recruit dendritic cells (Figure 3A).

The immunofluorescence technique was employed to label the predominant immune cells within the tumor microenvironment, including dendritic cells, tumor‐associated macrophages, tumor‐associated neutrophils, CD8^+^ T cells, and CD4^+^ T cells. We observed that at the interface between tumor necrosis and residual tumor in specimens treated with ILvax, there was an increase in the infiltration of dendritic cells and CD8^+^ T cells, while the number of neutrophils exhibited a slight decreas, which aligned with the observed trends in chemokine levels (Figure 3B, C). Further analysis of dendritic cell subsets indicated that the infiltration of dendritic cells induced by ILvax administration was predominantly comprised of CD103^+^ cDC1 (Figure 3D–F).

The migration of antigen‐presenting dendritic cells into peripheral lymphoid organs represents a crucial intermediary step in the facilitation of the cancer immune cycle. Flow cytometry analysis of the tumor‐draining lymph nodes and spleens from tumor‐bearing mice treated with ILvax demonstrated a significant increase in CD103^+^ cDC1, while CD11b^+^ cDC2 exhibited a notable decrease (Figure 3G, J). The results obtained from immunofluorescence staining for CD11c and Clec9a markers in the draining lymph nodes and spleens corroborated this observation (Figure S3A, B). In addition, the increased expression of SIINFEKL‐H2kb and CCR7 in CD103^+^ cDC1 detected by flow cytometry indicates the enhanced antigen‐presenting and migratory capabilities of CD103^+^ cDC1 in the treatment group (Figure 3H, I, K and L).

We established an in vitro co‐culture system comprising KPC‐OVA/KPC‐GFP, CD103^+^ cDC1 and OT‐I mouse CD8^+^ T cells to validate the generation of the cancer immune cycle induced by ILvax administration (Figure S3E). First, we observed that CD103^+^ cDC1 exhibited a greater capacity to phagocytize KPC‐GFP treated with ILvax, leading to an increased proportion of GFP^+^ CD103^+^ cDC1 (Figure S3D). In addition, we observed that CD103^+^ cDC1 co‐cultured with ILvax‐treated KPC‐OVA exhibited a heightened capacity to activate OT‐I mouse CD8^+^ T cells with increased expression of CD69 and CD25 (Figure S3F, G), as well as enhanced proliferation of these T cells with increased expression of Ki‐67 and decreased expression of CFSE (Figure S3H, I). Elisa analysis of IFN‐γ and TNF‐α in co‐culture system supernatant corroborated the activation of OT‐I CD8^+^ T cells in group 4 (G4) (Figure S3J, K).

To determine whether cDC1 serves as the effector cell that activates systemic anti‐tumor responses by ILvax administration, we systematically depleted cDC1 using anti‐Clec9a antibodies and depleted macrophages using anti‐F4/80 antibodies (Figure 3M). As is customary, compared with 6/6 in the distal model and 6/6 in the liver metastasis model in the IgG group, the incidence of rechallenge rate was reduced to 1/6 in the distant model and 1/6 in the liver metastasis model in ILvax group. However, under conditions of systematic cDC1 depletion induced by anti‐Clec9a antibody, the activation of systemic anti‐tumor responses induced by ILvax administration was inhibited, leading to a recovery of rechallenge rate of 6/6 in both the distal model and the liver metastasis model (Figure 3N–P and Figure S3C). In contrast, when macrophages were systematically depleted using anti‐F4/80 antibody, the rechallenge rate remained relatively stable, with 2/6 in the distal model and 1/6 in the liver metastasis model.

After ILvax localized administration of subcutaneous KPC‐OVA tumors in immunocompetent mice, with systematically depleted cDC1 and macrophages, the generation of antigen‐specific CD8 T cells was detected by flow cytometry in primary tumors, peripheral lymphoid organs, including tumor‐draining lymph nodes and spleen, and peripheral blood of each group. Depletion of systemic cDC1 restricts the expansion of SIINFEKL tetramer^+^ CD8^+^ T cells elicited by localized ILvax treatment of primary KPC‐OVA tumors (Figure 3Q–T). IFN‐γ enzyme‐linked immunospot (Elispot) analysis of isolated CD8 T cells from tumor drainage lymph nodes revealed a similar phenomenon that expansion of SIINFEKL tetramer^+^ CD8^+^ T cells was restricted under cDC1 depletion (Figure 3U, V). The clearance of cDC1 inhibited the movement of tumor antigens from tumors to peripheral lymphoid organs, leading to the disruption of cancer immune circle. According to the above results, we concluded that cDC1, rather than macrophages, serves as the effector cell that initiates the cancer immune cycle through ILvax administration, thereby activating systemic anti‐tumor responses. More importantly, the clearance of cDC1 significantly diminished the efficacy of the abscopal effect induced by ILvax localized administration of the primary tumors in combined with anti‐PD‐1 therapy for treating contralateral and liver metastatic tumors (Figure S3L–P). ILvax ablation enhances the tumor specific antigen‐cDC1‐endogenous CD8^+^ T cells axis, activating systemic anti‐tumor responses, thereby increasing the efficacy of immunotherapy on metastatic tumors.

#### 2.1.4 cDC1‐Mediated Antigen Spreading via ILvax Elicits Adoptive TCR‐T Therapy

The limited expansion and persistence of TCR‐T cells in vivo, coupled with the intrinsic characteristics of solid tumors, such as antigenic heterogeneity and immunosuppressive microenvironment significantly hinder the efficacy of adoptive T cells therapy for pancreatic cancer.

**FIGURE 4:**
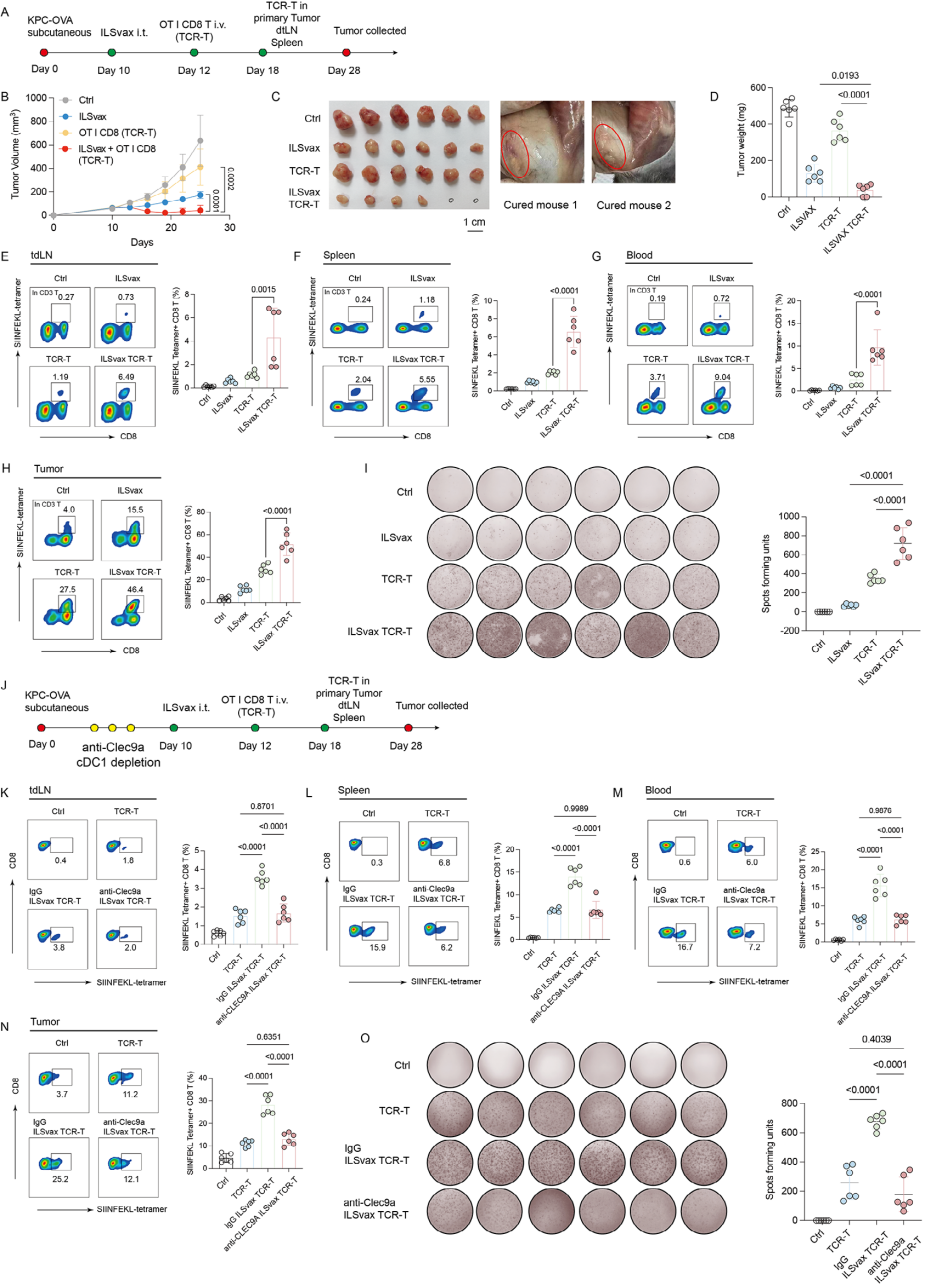
cDC1‐mediated antigen spreading via ILvax elicits adoptive TCR‐T therapy. (A) Illustration of ILvax and TCR‐T combination therapy in KPC‐OVA tumor‐bearing C57BL/6 mice. (B) Tumor growth curves in different groups, n = 6. Data are presented as mean ± SD, p value were determined by two‐way ANOVA with Tukey's post–hoc test. (C) The image of harvested tumors and cured mice after indicated administration. (D) The weight of harvested tumors from mice after indicated administration, n = 6. (E, F, G and H) Representative flow cytometry plots and quantitative analysis of SIINFEKL tetramer^+^ CD8^+^ T cells in tumors, tumor draining lymph nodes (dtLN), spleens and Peripheral blood, n = 6. (I) Images of IFN‐γ spot formed by dtLN CD8^+^ T cells after ex vivo re‐stimulation with SIINFEKL peptide, n = 6. (J) Illustration of ILvax and TCR‐T combination therapy in KPC‐OVA tumor‐bearing C57BL/6 mice received cDC1 depletion. (K, L, M and N) Representative flow cytometry plots and quantitative analysis of SIINFEKL tetramer^+^ CD8^+^ T cells in tumor, tumor draining lymph nodes (dtLN), spleens and peripheral blood, n = 6. (O) Images of IFN‐γ spot formed by dtLN CD8^+^ T cells after ex vivo re‐stimulation with SIINFEKL peptide, n = 6. Data are presented as mean ± SD and p value were determined by one‐way ANOVA with Tukey's multiple comparisons test (D, E, F, G, H, I, K, L, M, N, and O), Source data are provided as a Source Data file.

By alleviating the intrinsic characteristics of immune suppression and activating the cancer immune cycle, ILvax administration of primary tumor facilitated the infiltration and expansion of adoptive T cells within the tumor, as demonstrated by in vivo imaging technology, which enables the real‐time monitoring of fluorescence‐labeled TCR‐T cells within tumors (Figure S4A–C). Fluorescence imaging of tumor tissues confirmed the infiltration and expansion of TCR‐T cells (Figure S4D). To further explore whether the activation of systemic anti‐tumor responses induced by ILvax administration of primary tumor enhances the expansion and persistence of TCR‐T cells in vivo, thereby confirming the inhibitory effect of adoptive T cells therapy on pancreatic cancer, OT‐I CD8^+^ T cells as TCR‐T targeting antigen ovalbumin were combined with ILvax administration to treat KPC‐OVA subcutaneous tumors in immunocompetent mice (Figure 4A). The volume of subcutaneous tumors in mice treated with the combination of the ILvax administration and TCR‐T was only 1/4 of that in mice that received ILvax monotherapy and 1/6 of that in mice that received TCR‐T monotherapy. (ILvax vs. ILvax combined with TCR‐T, *p* = 0.0001, TCR‐T vs. ILvax combined with TCR‐T, *p* = 0.0002) (Figure 4B), and the weight was 1/3 of that in mice that received ILvax monotherapy and 1/5 of that in mice that received TCR‐T monotherapy (ILvax vs. ILvax combined with TCR‐T, *p* = 0.0193, TCR‐T vs. ILvax combined with TCR‐T, *p* < 0.0001) (Figure 4D). Excitingly, 1/3 of the mice in the combination therapy group achieved a complete response (CR) (Figure 4C). The observed enlargement of tumor‐draining lymph nodes and the spleen indicates a proliferation of TCR‐T cells within peripheral lymphoid organs (Figure S4E–G). We further assessed the proportion of adoptive TCR‐T cells in lymphoid organs, peripheral blood and primary tumors using flow cytometry (Figure S4H). Following ILvax administration of the primary KPC‐OVA tumors, we observed a significant increase of TCR‐T cells within lymphoid organs and peripheral blood (TCR‐T vs. ILvax combined with TCR‐T in tdLN *p* = 0.0015, in Spleen *p* < 0.0001, and in peripheral blood *p* < 0.001) (Figure 4E–G). The IFN‐γ enzyme‐linked immunospot (Elispot) assay performed on CD8^+^ T cells isolated from tumor‐draining lymph nodes, following stimulation with the ovalbumin, further confirmed the expansion of TCR‐T cells subsequent to ILvax administration of KPC‐OVA primary tumors (Figure 4I). In addition, ILvax administration enhances the proliferation of TCR‐T cells within tumors. The flow cytometry analysis showed that the proportion of TCR‐T cells among CD8 T cells in the ILvax combined with TCR‐T group is approximately 1.8 times greater than that observed in the TCR‐T monotherapy group (Figure 4H). cDC1 functions as the effector cell in activating systemic anti‐tumor responses. To explore whether the enhancement of the tumor specific antigen‐cDC1‐exogenous CD8^+^ T cell axis mediated by ILvax promotes the expansion of adoptive T cells, we systematically depleted cDC1 population via anti‐Clec9a antibody before treatment (Figure 3J). The depletion of cDC1 results in the disruption of the cancer immune cycle, thereby inhibiting the proliferation of adoptive T cells within lymphoid organs and peripheral blood (Figure 4K–M). The IFN‐γ enzyme‐linked immunospot (Elispot) assay performed on CD8^+^ T cells isolated from tumor‐draining lymph nodes, following stimulation with the ovalbumin, further confirmed the inhibition of TCR‐T cells expansion subsequent to ILvax administration of KPC‐OVA primary tumors (Figure 4O). In addition, the clearance of cDC1 impeded the expansion effect of intratumoral TCR‐T cells (Figure 4N). The disruption of the cancer immune cycle initial step, antigen spreading, hampers the expansion and persistence of adoptive T cells in vivo, consequently diminishing the efficacy of the ILvax and TCR‐T combination therapy (Figure S4I–K).

#### 2.1.5 ILvax Ablation Induces Cell‐Intrinsic Enhancements in Adoptive T Cells Functionality

**FIGURE 5:**
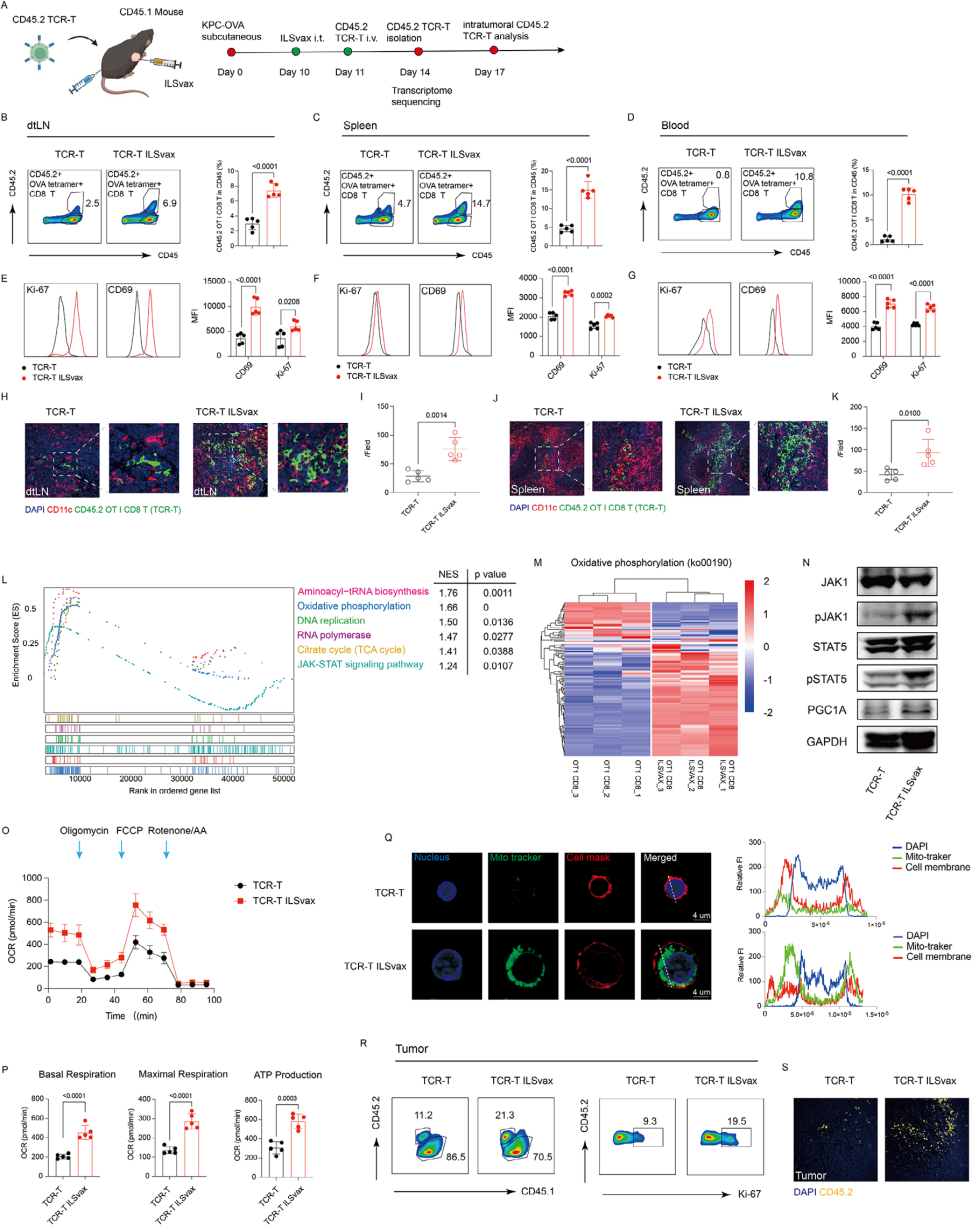
ILvax ablation induces cell‐intrinsic enhancements in adoptive T cells functionality. (A) Tumor‐bearing CD45.1mice received CD45.2 TCR‐T and ILvax treatment, TCR‐T cells were isolated from tumor and dtLN for RNA‐seq. (B, C and D) Representative flow cytometry plots and quantitative analysis of CD45.2 TCR‐T in tumor draining lymph nodes (dtLN), spleens and peripheral blood after the indicated treatment. (E, F and G) Representative histogram and quantitative analysis of Ki‐67 and CD69 in CD45.2 TCR‐T after the indicated treatment. (H and J) Representative images showing IF‐stained CD11c and CD45.2 in dtLN and spleen. (I and K) Quantitative analysis of CD45.2 TCR‐T in dtLN and spleen in different groups (L) GSEA showing enriched pathways in ILvax boosted CD45.2 TCR‐T cells. (M) Heatmap of oxidative phosphorylation related genes expression of CD45.2 TCR‐T after the indicated treatment via transcriptome analysis. (N) Immunoblot analysis of Jak1, p‐Jak1, Stat5, p‐Stat5 and Pgc1‐α in CD45.2 TCR‐T after the indicated treatment. (O). Oxygen Consumption Rate (OCR) curves of CD45.2 TCR‐T isolated from CD45.1 mice in in different groups. (P) Quantitative analysis basal respiration, maximal respiration and ATP production in CD45.2 TCR‐T. (Q) Representative images of mitochondrial within CD45.2 TCR‐T. (R) Representative flow cytometry plots and quantitative analysis CD45.2 TCR‐T and Ki‐67^+^ CD45.2 TCR‐T in tumors. (S) Representative IF images of CD45.2 TCR‐T in tumors. Data are presented as mean ± SD and p value were determined by two‐tailed unpaired student's t‐test (B, C, D, E, F, G, I, K, and P). Source data are provided as a Source Data file.

We subsequently aimed to elucidate the mechanisms by which ILvax boosting enhances exogenous T cells priming. Initially, we investigated whether the anti‐tumor efficacy of ILvax boosting was simply attributable to an increase in the number of TCR‐T cells or to modifications in TCR‐T cells functionality. CD45.2 TCR‐T cells were transferred into tumor‐bearing CD45.1 mice, following localized ILvax administration of primary tumors or in the absence of any treatment (as control). 6 days later, CD45.2 TCR‐T cells in tdLN were isolated for bulk RNA‐seq (Figure 5A). Before bulk RNA‐seq, we assessed the expansion of CD45.2 TCR‐T cells in vivo following ILvax administration of primary tumors. The proportion of CD45.2 TCR‐T cells in total immune cell population increased, with 3% in TCR‐T group vs. 7% in TCR‐T and ILvax combination group in dtLN, 5% in TCR‐T group vs. 15% in TCR‐T and ILvax combination group in Spleen, and 1% in TCR‐T group vs. 10% in TCR‐T and ILvax combination group in peripheral blood (Figure 5B–D). Immunofluorescence staining for CD45.2 maker in tumor‐draining lymph nodes and spleens further validated the expansion of TCR‐T cells within peripheral lymphoid organs (Figure 5H–K). In addition, compared to the TCR‐T group, the expression of proliferation marker Ki‐67 and activation marker CD69 on CD45.2 TCR‐T cells were significantly elevated in TCR‐T and ILvax combination group (Figure 5E, F and J).

In the context of elucidating the amplification of TCR‐T, CD45.2 TCR‐T were isolated from two groups for bulk RNA‐seq to verify the changes of intrinsic functions. PCA analysis demonstrates a strong homogeneity among the samples (Figure S5A). Our RNA‐seq data revealed that ILvax administration of primary tumors significantly altered the CD45.2 TCR‐T transcriptome profile, with the expression of 1944 genes upregulated and that of 9656 genes downregulated (Figure S5B). Gene set enrichment analysis (GSEA) of isolated TCR‐T cells revealed that ILvax‐boosted TCR‐T cells maintained a high proliferative potential, as evidenced by elevated DNA replication and JAK‐STAT signaling pathway target genes (Figure 5L, Figure S5C, D). ILvax‐boosted TCR‐T cells also exhibited a notable upregulation of metabolic pathways, including oxidative phosphorylation (OXPHOS) and the tricarboxylic acid (TCA) cycle (Figure 5L, M, Figure S5C, E). Prompted by these transcriptional signatures, we verified the activation of the Jak‐Stat5 pathway. More importantly, we conducted an analysis of the intracellular expression of peroxisome proliferator‐activated receptor‐gamma coactivator (PGC)‐1α, a master transcription factor (TF) that regulates numerous genes and pathways associated with oxidative phosphorylation (OXPHOS), and found that ILvax‐boosting led to an increase in PGC‐1α levels within TCR‐T cells (Figure 5N). PGC‐1α plays a crucial role in the generation and maintenance of mitochondria, and we observed elevated levels of mitochondria in TCR‐T cells that were boosted with ILvax (Figure 5Q). Seahorse analysis of the oxygen consumption rate (OCR) demonstrated an enhancement in mitochondrial oxidative phosphorylation within ILvax‐boosted TCR‐T cells (Figure 5O, P). To verify that the TCR‐T with enhanced intrinsic functions eliminated tumor cells effectively, TCR‐T with or with not ILvax boosted were isolated to kill tumor cells in vitro (Figure S5F, G). This approach revealed that, even when the same number of TCR‐T cells were present, ILvax‐boosted TCR‐T cells exhibited enhanced tumor cytotoxicity, suggesting that ILvax boosting improves the intrinsic functionality of TCR‐T cells (Figure S5H–M).

Suppressive immune microenvironment imposes constraints on the proliferation and cytotoxic function of adoptively transferred TCR‐T cells within the tumor microenvironment. The modulation of tumor immune microenvironment and the activation of cancer immune cycle by ILvax facilitate the infiltration and proliferation of adoptive TCR‐T cells within tumors (Figure 5R, S), while also exhibiting enhanced cytotoxicity (Figure S5O). In addition, TCR‐T and ILvax combination therapy alleviated the cytotoxicity of endogenous CD8 T cells (Figure S5P).

Therefore, the anti‐tumor efficacy of ILvax boosting was attributable to the increase in TCR‐T cell numbers and modification in TCR‐T cell functionality simultaneously.

#### 2.1.6 Localized ILvax Administration Combined with TCR‐T and anti‐PD‐1 Immunotherapy to Eliminate Pancreatic Cancer

**FIGURE 6:**
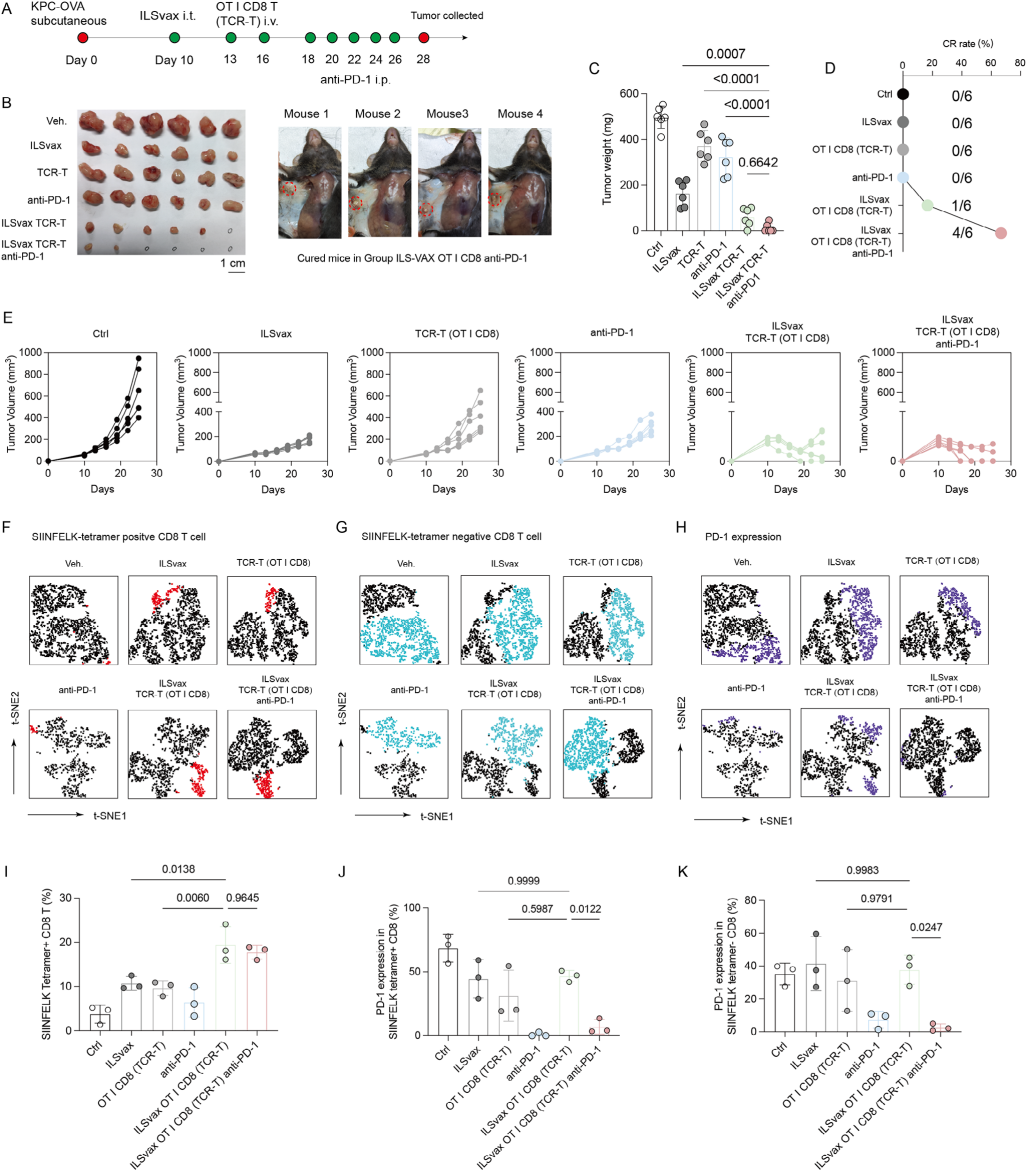
ILvax ablation combined with TCR‐T and anti‐PD‐1 immunotherapy to eliminate pancreatic cancer. (A) Illustration of ILvax, TCR‐T and anti‐PD‐1 combination therapy in KPC‐OVA tumor‐bearing C57BL/6 mice. (B) The image of harvested tumors and cured mice after indicated administration. (C) The weight of harvested tumors from mice after indicated administration, n = 6. (D) CR rate in different groups. (E) Individual tumor growth curves in different groups. (F and I) Representative t‐SNE plots and quantitative analysis of SIINFEKL tetramer^+^ CD8^+^ T cells in tumors after indicated administration. (G) Representative t‐SNE plots SIINFEKL tetramer^−^ CD8^+^ T cells in tumors after indicated administration. (H, J and K) Representative t‐SNE plots of PD‐1 maker and quantitative analysis of PD‐1 expression in SIINFEKL tetramer^+^ CD8^+^ T cells and SIINFEKL tetramer^−^ CD8^+^ T cells. Data are presented as mean ± SD and p value were determined by one‐way ANOVA with Tukey's multiple comparisons test (D, I, J, and K). Source data are provided as a Source Data file.

The combination of ILvax administration and adoptive TCR‐T therapy effectively controlled tumor progression by activating both the endogenous and exogenous cancer immune cycles. However, complete tumor eradication was not achieved, potentially due to the exhaustion of adoptive T cells within the tumor microenvironment. Therefore, we conducted a triple‐combination therapy experiment utilizing ILvax, TCR‐T, and anti‐PD‐1 antibody to treat pancreatic cancer (Figure 6A). The triple‐combination therapy significantly controlled tumor progression (Figure 6B, C and E) and markedly increased the rate of complete response (CR). Specifically, 4/6 mice in the triple‐combination group achieved complete response, compared to 1/6 in the ILvax administration and TCR‐T combination group (Figure 6D).

Flow cytometry analysis revealed that the proportion of TCR‐T cells within tumors did not significantly increase in the triple‐combination group compared to ILvax administration and TCR‐T combination group (Figure 6F, I). However, the PD‐1 expression both on antigen‐specific T cells and antigen‐non‐specific T cells was significantly decreased in the triple‐combination group, which means anti‐ PD‐1 immunotherapy reduced exhausted TCR‐T cells and endogenous T cells (Figure 6G, H, J and K).

Therefore, anti‐PD‐1 immune checkpoint blockade significantly enhance the efficacy of ILvax and TCR‐T combined therapy in pancreatic cancer, ultimately increase the complete response of this combined therapy for treating pancreatic cancer.

## 3 Discussion

Here, we developed an ablation‐based in situ vaccine, ILvax, that effectively destroys pancreatic cancer while eliciting immune responses in the immune‐suppressive microenvironment. Unlike conventional vaccines, our ILvax stimulated the release of tumor‐derived self‐antigens, while facilitating the antigen spreading via CD103^+^ cDC1 cells, therefore leading to the amplified antigen‐specific CD8^+^ T cell responses. Previous work suggested that antigen presentation via dendritic cells (DCs) majorly contributes to the vaccination‐like responses [14, 27, 28]. Our work further identified the responsible DC subpopulation, namely the CD103^+^ cDC1 cells, but not other DC subpopulations, that serve as a crucial determinant in generating vaccination‐like responses after ILvax administration.

Antigen presentation associated CD103^+^ cDC1 cells, followed by IL administration, enhance the expansion of adoptive TCR‐T cells, while concurrently enhancing TCR‐T cells metabolism and polyfunctionality, which exhibited significant synergism in combating pancreatic cancer. It is the first time that a mechanism explaining how locoregional therapy remodulates adoptive T cells was proposed.

Targeting the cancer immune cycle holds great potential for an in situ vaccine [29, 30, 31]. It emphasizes the iterative nature of the immune response, wherein the elimination of tumor cells by T cells initiates subsequent rounds of antigen release, antigen presentation and T cell stimulation, maintaining active immunity and adapting it to tumor evolution. The characteristic of low mutational burden in pancreatic cancer significantly restricts the initiation step of the cancer immune cycle, antigen release and presentation, therefore interrupting the iterative nature of the cancer immune cycle and failing to maintain a sustained anti‐tumor immunity, ultimately resulting in the poor therapeutic efficacy and prognosis [32, 33, 34]. The disruption of the cancer immune cycle is primarily attributed to the deficiency of tumor‐specific antigens or the inadequacy of therapeutic interventions in releasing tumor‐specific antigens. Previous studies have demonstrated that locoregional therapy may augment the cancer immune cycle in immune‐suppressive tumors, therefore generating vaccine‐like responses [35, 36]. However, a clear mechanism of augmented vaccine‐like response remains to be elucidated. In our work, we have found that ILvax administration of pancreatic cancer drives the first step in the cancer immune cycle, that is, the spreading of tumor‐specific antigens via CD103^+^ cDC1. Subsequently, our study also demonstrated that the second step in the cycle drives antigen‐specific T cell expansion in a CD103^+^ cDC1‐dependent manner. Accordingly, the cDC1‐dependent activation variesand ultimately enhances the process of the cancer immune cycle.

Poor expansion and short‐lived persistence of adoptive T cells in vivo significantly limit the effectiveness of ACT treatment for solid tumors [37]. Several strategies have been proposed to enhance its long‐lasting efficacy, like introducing adjuvants such as cytokines, vaccines, and oncolytic viruses [38]. Vaccines can be specifically designed to target CAR T cells, thus enhancing their activation, proliferation, and persistence. Amph‐ligand, a model vaccine, has been applied to boost massive CAR‐T expansion, increasing donor cell polyfunctionality, and enhancing antitumor efficacy in multiple immunocompetent mouse tumor models [39]. Multiple clinical trials are exploring the feasibility of using CAR T cells in conjunction with vaccine therapy. A phase 1/2 clinical trial evaluated CAR T cells targeting the oncofetal antigen Claudin 6 (CLDN6) in solid tumors, utilizing an RNA vaccine to augment CAR T cell activity [22]. Preliminary data indicate manageable side effects and promising efficacy, with an objective response rate of 33% and a disease control rate of 67% among 21 evaluable patients. These findings underscore the potential of cancer vaccines to enhance the persistence and therapeutic impact of CAR T cells. Evgin et al. demonstrated that stimulation of the native T cell receptor (TCR) with viral or virally encoded epitopes leads to enhanced proliferation, improved CAR‐directed antitumor activity, and the development of distinct memory phenotypes in adoptive CAR‐T cells [23]. Clinical trials are currently underway to evaluate the efficacy of combining oncolytic viruses with adoptive T‐cell therapy in treating solid tumors. For instance, the NCT03740256 trial is assessing the combination of oncolytic adenovirus and HER2‐specific CAR T cells. Previous studies have shown that vaccine boost increases the number of adoptive T cells in peripheral blood by 3 to 6 times [39]. Incomparison, our results have revealed an amplification efficiency of 12 times for the number of adoptive T cells boosted by the IL vaccine. Accordingly, in our work, we demonstrated that localized administration of primary pancreatic cancer significantly enhances the in vivo expansion of adoptive TCR‐T cells, while concurrently enhancing adoptive TCR‐T cells metabolism and polyfunctionality in a CD103^+^ cDC1‐dependent manner, thereby resulting in enhanced tumor‐killing efficacy. We report for the first time that localized administration of tumors promotes the expansion and polyfunctional enhancement of adoptive T cells.

It is crucial to acknowledge and emphasize the limitations of the study as well. First, we applied OVA as a generalized model antigen, which could not fully represent the heterogeneous TAAs. Our results based on the OVA‐specific immune responses might introduce bias, distinguished from the real immune settings. Second, Clec9a antibodies deplete all cDC1 subpopulations, including the infiltrating CD103^+^ cDC1 and the tissue resident CD8α^+^ cDC1, suggesting CD8α^+^ cDC1, though with a very low proportion inside the tumors, might also contribute to ILvax‐induced antigen spreading. Finally, precise administration of ILvax to the central location in the tumor requires an interventional approach that poses a technical challenge for clinical application.

In conclusion, for the first time, we have demonstrated that ILvax locoregional ablation therapy for pancreatic tumors not only activates the endogenous cancer immune cycle via antigen spreading but also significantly enhances the functionality of exogenous adoptive T cells. The synergetic mechanism leads to sustained anti‐tumor immune response, offering a novel strategy to enhance adoptive cell therapy, currently limited in most clinical scenarios.

## 4 Methods

### 4.1 Ethics Statement

All procedures involving animals were performed in accordance with the guidelines of the Animal Ethics Committee of the First Affiliated Hospital of Zhejiang University School of Medicine. The mice under study were reared in a specific‐pathogen‐free (SPF) environment at the Experimental Animal Center, First Affiliated Hospital, School of Medicine, Zhejiang University. The ambient temperature was maintained at 21°C–22°C, with humidity levels ranging between 48% and 52%. The mice were subjected to a conventional 12:12 light/dark cycle, with lights being turned on at 6:00 a.m. and turned off at 6:00 p.m. The animal experiment was approved by the Institutional Animal Care and Use Committee of the First Affiliated Hospital Zhejiang University School of Medicine, approval No. 2026‐123. No procedure involving human specimens in this study.

### 4.2 ILvax Synthesis and ^1^H NMR Characterization

ILvax was synthesized via an ionic metathesis reaction between choline bicarbonate and geranic acid at a molar ratio of 1:1, creating an ILvax, as previously reported. ^1^H NMR characterization was performed and was found to be consistent with the previously published spectra.

### 4.3 Cell Lines and Cell Culture

The KPC cell line obtained from the LSL‐Kras G12D/+; LSL‐Trp53 R172H/+; Pdx1‐Cre mouse model, which was kindly provided by Prof. Raghu Kalluri (Department of Cancer Biology, Division of Basic Sciences, MD Anderson Cancer Center, Houston, TX, USA). PANC02 cell lines were purchased from the American Type Culture Collection (ATCC, Manassas, VA, USA). KPC was cultured in modified McCoy's 5A Medium (16600108, Thermo Fisher Scientific) with 10% FBS and 1% Pen‐Strep. PANC02 were cultured in high‐glucose Dulbecco's modified Eagle's medium (DMEM; 11965092, Gibco) supplemented with 10% fetal bovine serum (FBS) and 1% Penicillin‐Streptomycin (Pen‐Strep). All cell lines were incubated at 37°C under 5% CO_2_. Mycoplasma contamination was routinely evaluated by PCR.

Ovalbumin (OVA)‐ or luciferase (RLUC)‐ overexpressing lentiviruses containing the puromycin selection marker was used to generate OVA‐ (KPC‐OVA, Panc02‐OVA) or KPC‐LUC stablely‐expressed pancreatic cancer cells, respectively.

### 4.4 Animals

C57Bl/6 J mice (all male, aged 6–8 weeks) were obtained from the Model Animal Research Center of Nanjing University. The animals were maintained under specific‐pathogen‐free (SPF) conditions in the Experimental Animal Center at the First Affiliated Hospital, School of Medicine, Zhejiang University.

### 4.5 Animal Experiments

Mice were assigned randomly to either the control or experimental groups. For orthotopic tumor implantation, 25 µL of diluted Matrigel (Corning, Cat. #354248) containing 5 × 10^5^ KPC, KPC‐OVA, or KPC‐LUC cells were orthotopically injected into the tail of the pancreas. For subcutaneous tumor implantation, C57BL/6 mice were subcutaneously injected with 5 × 10^5^ KPC, KPC‐OVA cells into the right flank. Subcutaneous tumor growth was evaluated by measuring the tumors with a caliper and calculating the tumor volume as length×width 2 × 0.5. The maximal tumor size/burden permitted by our ethics committee review board was 2000 mm^3^. We confirm that none of the mice included in this study exceeded this limit. The mice were weighed before sacrifice. Tumors were harvested, weighed, and divided into segments for subsequent analysis.

The liver metastasis models were established using the semi‐spleen method. The spleens of the mice were first surgically exposed, and then the spleen was clamped from the middle with a 4 mm mini‐vascular clip, avoiding damage to the blood vessels on both sides of the spleen. A total of 200 µL saline containing 2 × 10^6^ cancer cells were steadily injected into the lower half of the spleen, followed by 200 µL of saline for perfusion and irrigation. The vessels at the hilum of the lower part of the spleen were ligated, and then the lower part of the spleen was cut off. The spleen was then inserted back into the abdominal cavity. Finally, the skin was sutured layer by layer.

### 4.6 The Bioluminescence Experiments

The in vivo bioluminescence experiments were performed using mice bearing KPC‐LCU orthotopic pancreatic tumors and liver metastasis. The tumor‐bearing mice were anaesthetised with isoflurane inhalation, and were injected with Luciferase substrate (100 mg/kg, i.p., D‐Luciferin, Goldbio, Cat. #115144‐35‐9). After 15 min of injection, the mice were put into the IVIS spectrum imager (IVIS Lumina LT, PerkinElmer) for bioluminescence analysis. The radiance intensity of region of interest was assessed using the Living Image Software (Living Imaging, PerkinElmer, version 4.2). The bioluminescence experiments were performed at the Department of Laboratory Animals, the First Affiliated Hospital, School of Medicine, Zhejiang University. The experiments were performed under the guidelines of Laboratory Animals Welfare and Ethics of the Institute.

### 4.7 In Vivo Administration of Antibody Drugs

CD8^+^ T, CD4^+^ T, NK Cells, cDC1 and Macrophage Depletion Were Achieved by Intraperitoneal injection of anti‐CD8α antibody ((InVivoMAb anti‐mouse CD8α, Clone: 2.43, Cat. # BE0061), anti‐CD4 antibody (InVivoMAb anti‐mouse CD4, Clone: GK1.5, Cat. #BE0003‐1), anti‐NK1.1 antibody (InVivoMAb anti‐mouse NK1.1, Clone: PK136, Cat. # BE0036), anti‐Clec9a antibody (InVivoMAb anti‐mouse CLEC9A, Clone: 7H11, Cat. #BE0305), and anti‐F4/80 antibody (InVivoMAb anti‐mouse F4/80, Clone: A3‐1, Cat. #BE0206).

For in vivo immunotherapies, the anti‐PD‐1 antibody (BioXCell, Clone: RMP1‐14, Cat. #BE0146) or the isotype control (BioXCell, Clone: 2A3, Cat. #BE0089) were injected intraperitoneally after ILvax and TCR‐T combination therapy.

### 4.8 OT‐I CD8 T Cells (TCR‐T) Isolation and TCR‐T therapy

OT‐I CD8^+^ T cells (TCR‐T) for treating OVA‐KPC tumors were isolated from the spleens of OT‐I mice using a magnetic bead sorting method (STEMCELL, EasySep Mouse Naive CD8^+^ T Cell Isolation Kit). For TCR‐T therapy, each KPC‐OVA tumor‐bearing mouse received an intravenous injection of 3 × 10^6^ TCR‐T cells via the tail vein.

### 4.9 CD103+ Dendritic Cells Isolation

CD103^+^ DCs were obtained from the spleens and lymph nodes of C57BL/6 mice by flow cytometry sorting.

### 4.10 SIINFEKL Tetramer Antibody Preparation for Flow Cytometry

The SIINFEKL tetramer antibody was prepared according to the Flex‐T Fixed Peptide Tetramer Preparation and Flow Cytometry Staining Protocol (BioLegend).

### 4.11 Flow Cytometry

KPC and KPC‐OVA tumors were mechanically dissociated and enzymatically digested in RPMI‐1640 medium supplemented with 2% FBS, 10 µg/mL DNase I (D5025, Sigma–Aldrich), 1 mg/mL collagenase IV (17104019, Thermo Fisher Scientific), and 3 mM CaCl2 (21115, Sigma–Aldrich) at 37°C for 40 min. The dissociated tissues were filtered through 70 µm strainers to obtain a single‐cell suspension. Non‐immune cells were depleted using a 36% Percoll solution. Invitrogen LIVE/DEAD Fixable Dead Cell Stain was used to distinguish dead cells, and live cells were further incubated with an anti‐CD16/CD32 Ab for 30 min to block Fc‐mediated reactions. Surface marker expression was analyzed by incubating the cells with specific antibodies in the dark at 4°C for 30 min. For intracellular cytokine detection, cells were stimulated with a Cell Stimulation Cocktail (550583, BD Biosciences) for 4 h. Cells intended for intracellular staining of Granzyme B, IFN‐γ, TNF‐α, and Ki67 were further processed using intracellular fixation and permeabilization kits (555028, BD Biosciences or 225870, Invitrogen) according to the manufacturer's protocols. Data acquisition was conducted on a Fortessa cell analyzer (BD Biosciences) or a Cytek Aurora (Cytek Biosciences), and data analysis was performed using FlowJo software (Becton Dickinson, version 10.8.1).

### 4.12 ELISA

Culture supernatants were individually collected. ELISA kits were used to quantify IFN‐γ and TNF‐α secreted from OT‐I mouse CD8^+^ T cells.

### 4.13 Western Blotting

RIPA lysis buffer (P0013B, Beyotime Biotechnology) supplemented with 1 mm phenylmethanesulfonyl fluoride (ST505, Beyotime Biotechnology) was used to lyse cells following centrifugation at 12 000 g for 15 min at 4°C. The protein concentration in the supernatant was determined after boiling for 5 min in 1× NuPAGE LDS sample buffer (Thermo Fisher Scientific). Proteins were separated by SDS‐PAGE using 8%–12% polyacrylamide gels and then transferred onto a PVDF membrane (IPFL00010, Millipore). The membrane was blocked with 5% skimmed milk in TBST for 1 h at room temperature and incubated overnight at 4°C with the indicated primary antibodies. After three washes with TBST, the membranes were incubated with HRP‐conjugated secondary antibodies for 1 h at room temperature and visualized using a ChemiScope Touch imaging system (Clinx Science Instruments).

### 4.14 Immunofluorescence Staining

Tissues were harvested and fixed in 10% buffered formalin phosphate for over 48 h. The fixed tissues were embedded in paraffin, and sliced into sequential 4 µm thick slides. Antigen retrieval was performed using sodium citrate buffer. Tissue sections were blocked with 3% BSA buffer for 30 min. After overnight incubation with primary antibodies, sections were treated with fluorescent‐conjugated secondary antibodies. Following staining, the sections were mounted with DAPI and mounted using an anti‐fade mounting medium. Fluorescent images were acquired using a TCS SP8 X confocal microscope (Leica).

### 4.15 Metabolic Studies

The metabolic activity of CD8+ T cells was assessed using a Seahorse XFe24 Analyzer (Agilent) to measure the oxygen consumption rate (OCR). Specifically, 1 × 10^5^ CD8^+^ T cells were seeded in Seahorse 24‐well plates, and the Seahorse XF Cell Mitochondrial Stress Test was performed according to the manufacturer's protocol. OCR measurements were conducted in XF RPMI1640 medium supplemented with 10 mm glucose, 2 mm L‐glutamine, and 1 mm sodium pyruvate. Basal OCR was measured, followed by sequential treatments with 2 µm oligomycin (to inhibit ATP synthesis), 1 µm FCCP (to uncouple oxidative phosphorylation from the electron transport chain), and a combination of 0.5 µm rotenone and antimycin A (to inhibit complexes I and III of the electron transport chain).

### 4.16 RNA Sequencing and Data Analysis

mRNA sequencing was performed on KPC tumors vs ILvax treated KPC tumors and TCR‐T cells vs ILvax boosted TCR‐T cells. Total RNA was extracted using the RNA‐Quick Purification Kit (RN001, YiShan Biotechnology). Library construction, sequencing (Illumina), and analysis were conducted at the Biomedical Big Data Center, First Affiliated Hospital, School of Medicine, Zhejiang University and Sangon Biotech (Shanghai) Co., Ltd.

### 4.17 Statistical Analysis

The Student's *t*‐test was used for comparisons between the two groups. One‐way analysis of variance (ANOVA) was used to compare multiple groups. The log‐rank test or the Gehan–Breslow–Wilcoxon test was used for survival analysis. Overall survival curves were analyzed using the Kaplan‐Meier method and compared with the log‐rank test. Statistical analysis was performed using SPSS 18.0 and GraphPad Prism 7.0. The results are presented as mean ± standard deviation (SD) of at least three independent experiments, and statistical significance was set at *P* < 0.05.

## Author Contributions

J.H., Q.H. and T.L. conceived and designed the project and contributed to the interpretation of data. J.H., Q.W., Y.Z., Qing.W., Y.J., M.Y., M.W., S.S., X.Y. and F.Z. contributed to the acquisition and analysis of data. J.H. drafted the manuscript. Q.H. and T.L. jointly supervised and revised the manuscript.

## Conflicts of Interest

The authors declare no conflict of interest.

## Supporting information




**Supporting File**: advs76135‐sup‐0001‐SuppMat.docx.

## Data Availability

The data that support the findings of this study are available in the supplementary material of this article.
