# Supplementary material for: Antigen Spreading via Localized Administration Enhances Adoptive TCR‐T Cell Therapy in Pancreatic Cancer
Source: Adv Sci (Weinh). 2026 Jun 19:e76135. Online ahead of print. doi: 10.1002/advs.76135 (PMC13336903; doi:10.1002/advs.76135)
Supplement: Supplementary file 1 — Supporting File: advs76135‐sup‐0001‐SuppMat.docx. [file ADVS-9999-e76135-s001.docx]

**Antigen spreading via localized administration enhance antitumor activity of adoptive TCR-T cells therapy in pancreatic cancer**

Junming Huang^1, 2, #^, Qin Wang^1, 2, #^, Zhuo Yao^1, 2^, Qing Wang^1, 2^, Yongtao Ji^1, 2^, Minqi Yang^1, 2^, Meng Wang^2, 6^, Shiyi Shao^1, 2^, Xinyu Zhao^1, 2^, Fu Zhang^1, 2^, Qida Hu^1, 2, 3, 4, 5, 6^ *, Tingbo Liang^1, 2, 3, 4, 5, 6^ *

**Supplemental information**

**Supplemental Figures and Legends**

Figure S1. ILvax ablation of pancreatic cancer modulates intratumoral immune microenvironment (related to Figure 1).

Figure S2. ILvax ablation of primary tumor elicits vaccine-like systemic effects (related to Figure 2).

Figure S3. Identification of cDC1 is indispensable for systemic anti-tumor responses of ILvax ablation (related to Figure 3).

Figure S4. cDC1-mediated antigen spreading via ILvax elicits adoptive TCR-T therapy (related to Figure 4).

Figure S5. ILvax ablation induces cell-intrinsic enhancements in adoptive T cells functionality (related to Figure 5).

**Supplemental Tables**

Table S1. Detailed information of antibodies for flow cytometry

Table S2. Detailed information of antibodies for western blotting

Table S3. Detailed information of antibodies for IF and IHC

Table S4. Detailed information of antibody drugs for immune cells depletion

Table S5. Detailed information of reagents used in this study


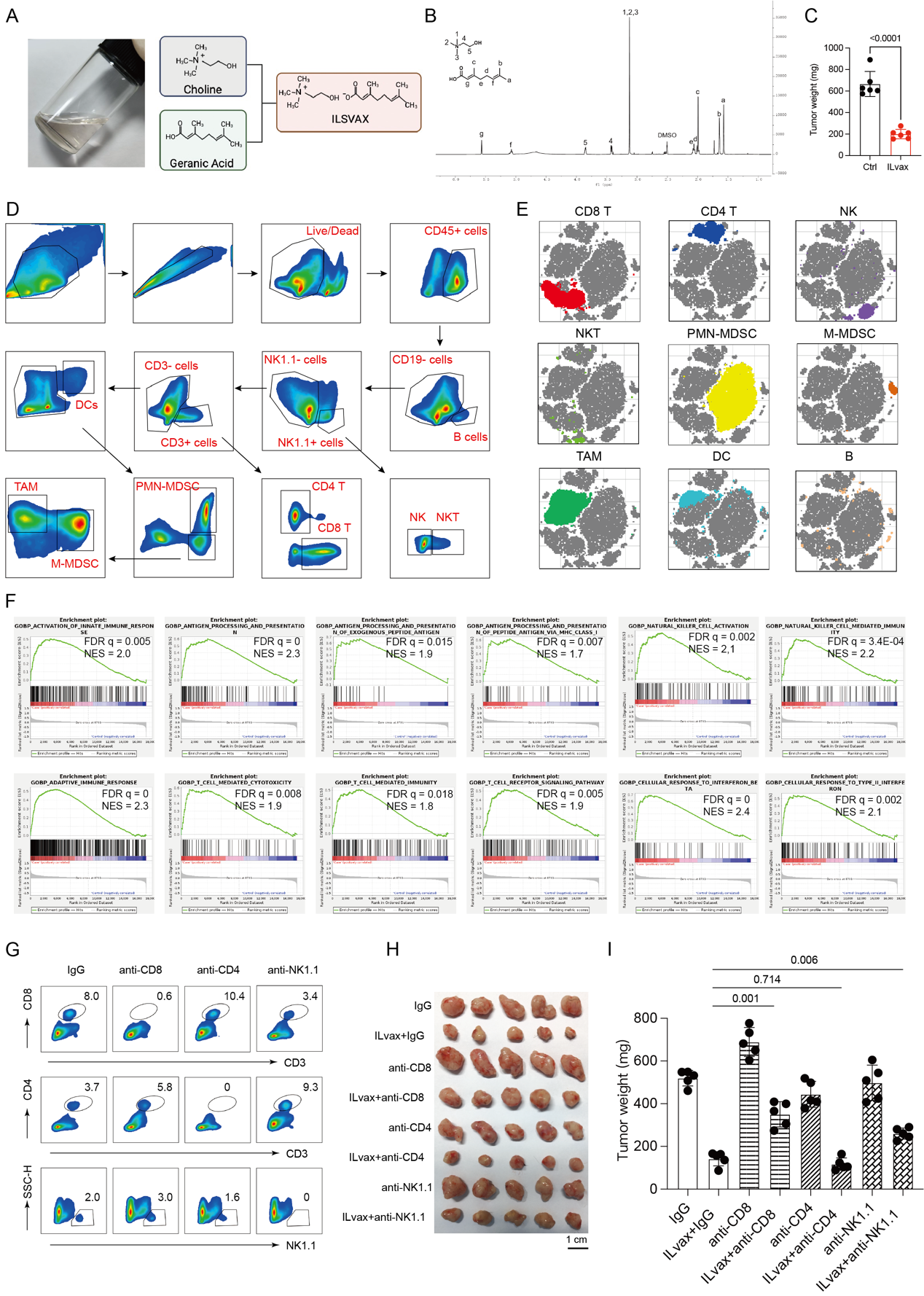


Figure S1. ILvax ablation of pancreatic cancer modulates intratumoral immune microenvironment (related to Figure 1)

(A) Chemical Structure of ILvax. (B) 1H NMR spectrum of ILvax. (C) Tumor weight of different groups, n=6. (D) Gating strategy for major immune cell populations in KPC orthotopic tumors. (E) t-SNE analysis of CD8^+^ T cells, CD4^+^ T cells, NK cells, NKT cells, PMN-MDSCs, M-MDSCs, TAMs, DCs, and B cells. (F) GSEA enrichment of DEGs between the PBS- and ILvax- treated tumours, including innate immune response, antigen processing and presentation, adaptive immune response, T cell mediated immunity, T cell mediated cytotoxicity, natural killer cell activation, natural killer cell mediated immunity, and cellular response to interferon-β. (G) The efficiency of anti-CD8, anti-CD4 and anti-NK1.1 monoclonal antibodies in eliminating CD8^+^ T cells, CD4^+^ T cells and NK1.1 cells was verified by flow cytometry. (H) The image of harvested tumours from mice after indicated administration. (I) Tumor weight of different groups, n=6. Data are presented as mean ± SD, p values were determined by two-tailed unpaired student’s t-test (C) and one-way ANOVA with Tukey’s multiple comparisons test (I). Source data are provided as a Source Data file.


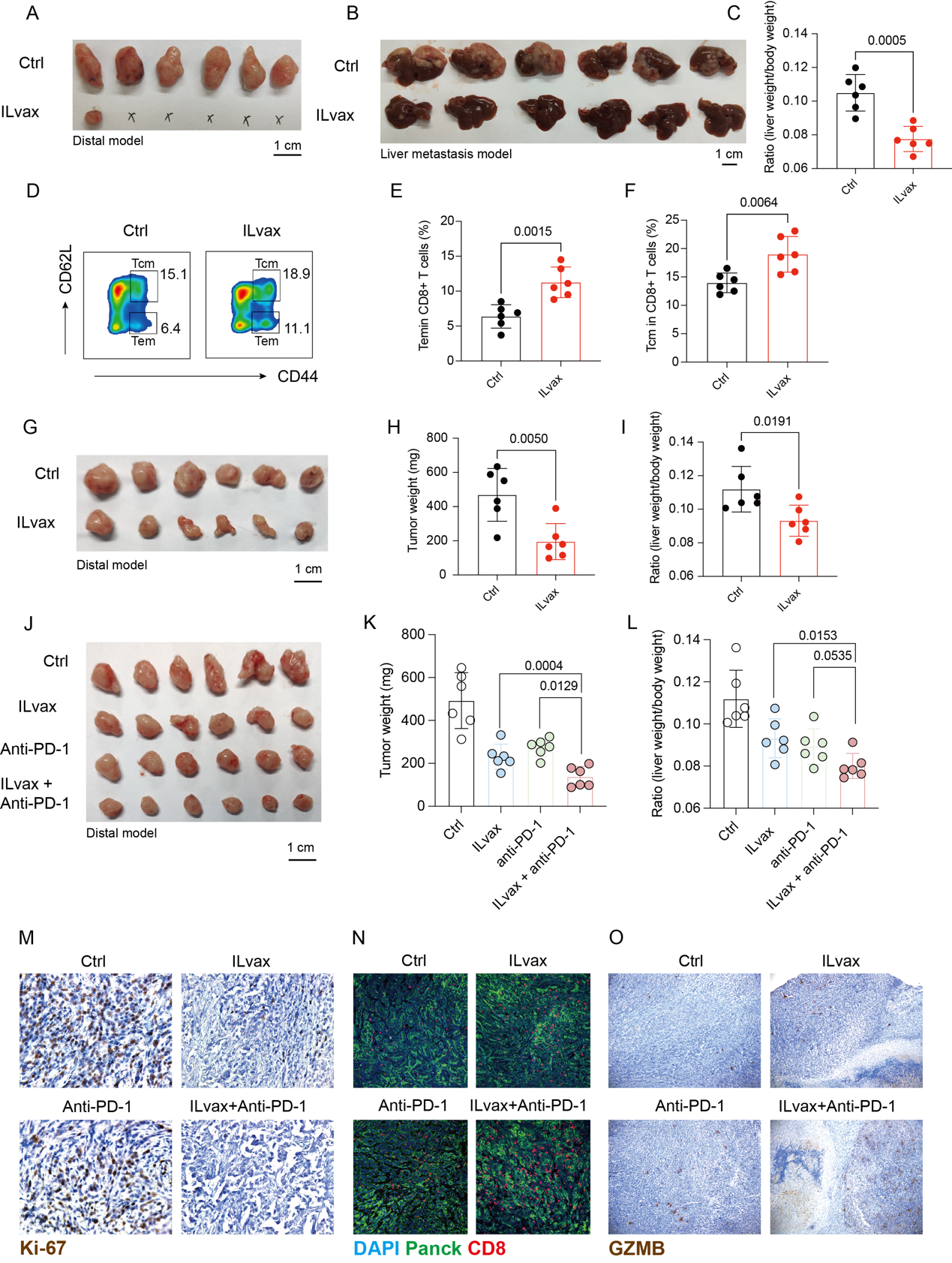


Figure S2. ILvax ablation of primary tumor elicits vaccine-like systemic effects (related to Figure 2).

(A) The image of harvested tumours in distal model from different groups. (B) The image of livers in liver metastasis model from different groups. (C) Liver-to-body weight ratio in different groups (n=6). (D) Representative flow cytometry plots and (E and F) quantitative analysis of CD44^+^ CD62L^+^ CD8^+^ T cells (Tcm) and CD44^+^ CD62L^-^ CD8^+^ T cells (Tem). (G) The image of harvested tumours in distal model after the indicated treatment. (H and I) Tumor weight in distal model and Liver-to-body weight ratio in liver metastasis model in different groups. (J) The image of harvested tumours in distal model after the indicated treatment. (K and L) Tumor weight in distal model and Liver-to-body weight ratio in liver metastasis model in different groups. (M) IHC of primary tumor Ki-67 expression after the indicated treatment. (N) IF of primary tumor CD8 expression after the indicated treatment. (O) IHC of primary tumor Granzyme B expression after the indicated treatment. Data are presented as mean ± SD. p values were determined by two-tailed unpaired student’s t-test (E, F, H, and I) and one-way ANOVA with Tukey’s multiple comparisons test (K, and L). Source data are provided as a Source Data file.


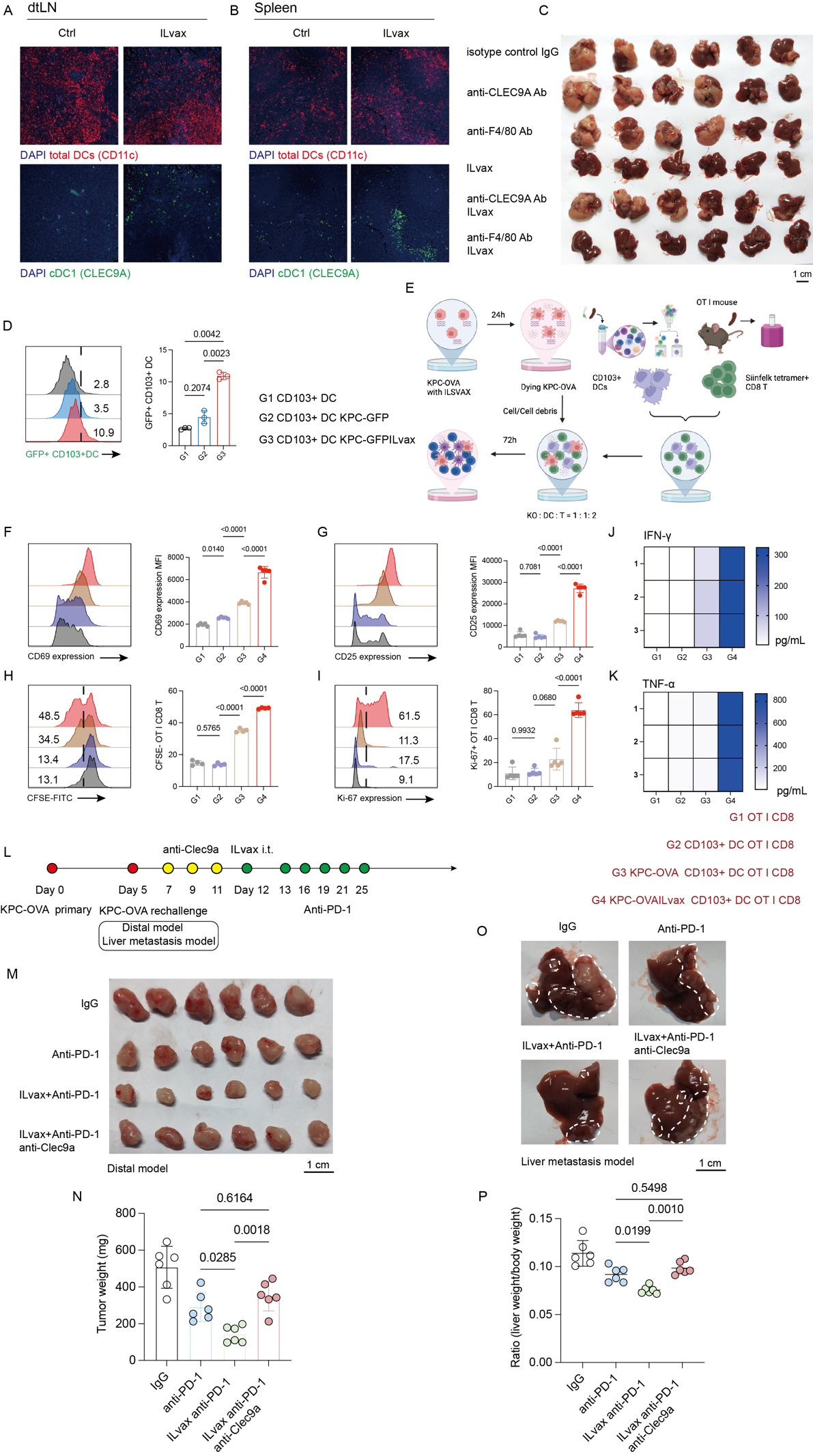


Figure S3. Identification of cDC1 is indispensable for systemic anti-tumor responses of ILvax ablation (related to Figure 3).

(A and B) cDC1 in dtLN and spleen after the indicated treatment of primary tumor. (C) The image of livers in liver metastasis model from different groups. (D) Representative histogram and quantitative analysis of GFP^+^ CD103^+^ DCs in different groups. (E) Schematic illustration of co-culture system consisting of KPC-OVA or ILvax-treated KPC-OVA, CD103^+^ DCs, and OT-I CD8^+^ T cells. (F and G) CD8^+^ T cells activation analysis, including CD69, and CD25 expression. (H and I) CD8^+^ T cells proliferation analysis, including CFSE and ki-67 expression. (J and K) ELISA kits were used to quantify IFN-γ and TNF-α secreted from OT-I mouse CD8 T cells after the indicated treatment, n=3. (L) Illustration of ILvax and anti-PD-1 combination therapy in rechallenge model. (M) The image of harvested tumours in distal model from different groups. (N) Tumor weight in different groups in distal model, n=6. (O) The image of livers in liver metastasis model from different groups. (P) Liver-to-body weight ratio in different groups, n=6. Data are presented as mean ± SD and p values were determined by one-way ANOVA with Tukey’s multiple comparisons test (D, F, G, H, I, N, and P). Source data are provided as a Source Data file.


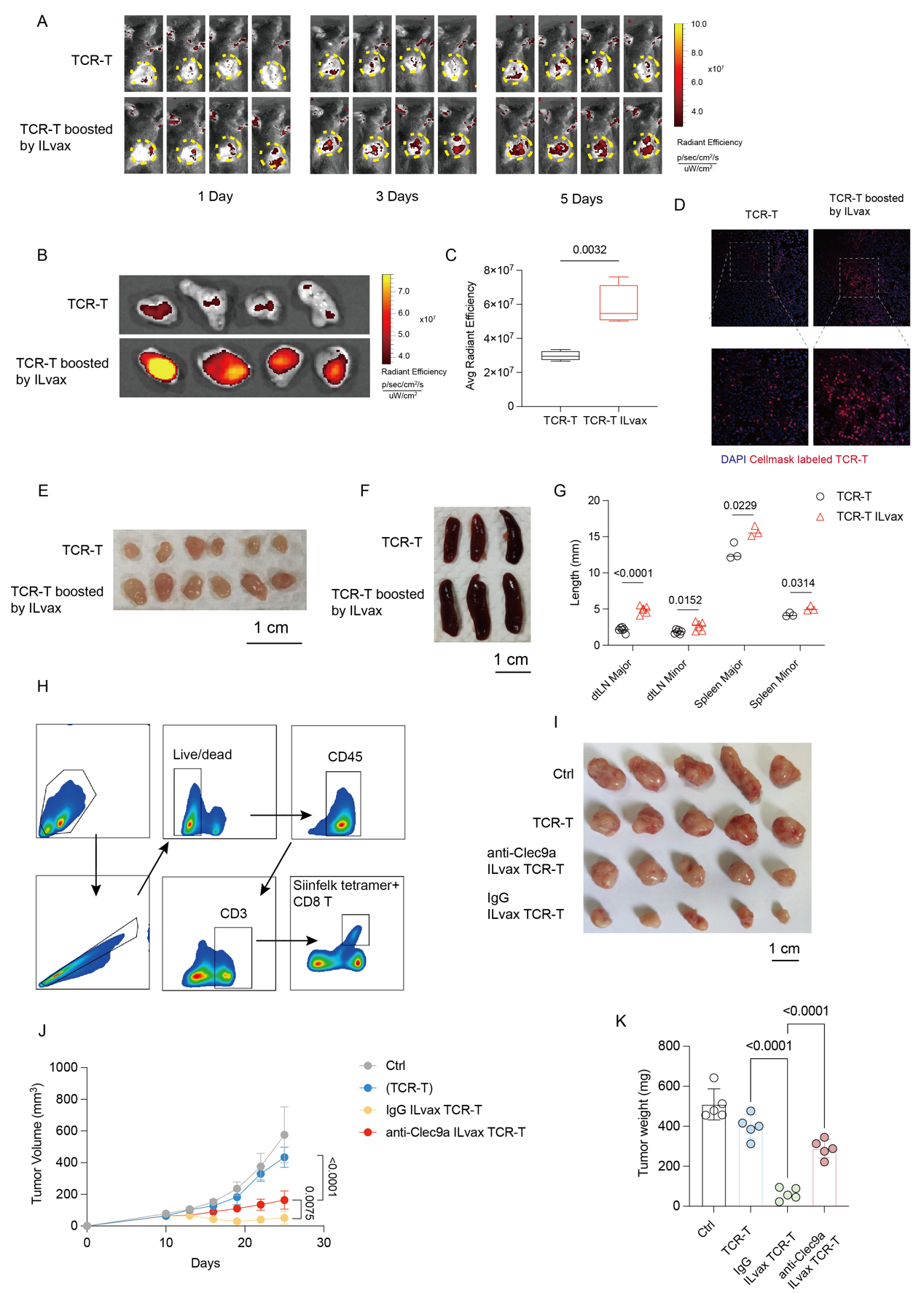


Figure S4. cDC1-mediated antigen spreading via ILvax elicits adoptive TCR-T therapy (related to Figure 4)

(A) In vivo intratumoral fluorescence monitor in day 1, 3, and 5 after fluorescence-labeled TCR-T injection in different groups. (B) The fluorescence imaging of harvested tumours in different groups. (C) Quantitative analysis of radiance intensity of harvested tumours in different groups, n=4. (D) Representative images of intratumoral fluorescence-labeled TCR-T via confocal imaging. (E) The image of harvested tumor-draining lymph nodes (tdLN) in different groups. (F) The image of harvested spleens in different groups. (G) Quantitative analysis of major and minor length of tdLN and spleen, n=6 in tdLN, n=3 in spleen. (H) Gating strategy for SIINFEKL tetramer^+^ CD8^+^ T cells. (I) The image of harvested tumours after the indicated treatment. (J and K) Tumour volume and tumor weight in different groups, n=6, log-rank (Mantel-Cox) test, *p* = 0.0075, anti-Clec9a+ILvax+TCR-T group v.s. IgG+ILvax+TCR-T group. Data are presented as mean ± SD. p values were determined by two-tailed unpaired student’s t-test (C and G) and one-way ANOVA with Tukey’s multiple comparisons test (K). Source data are provided as a Source Data file.


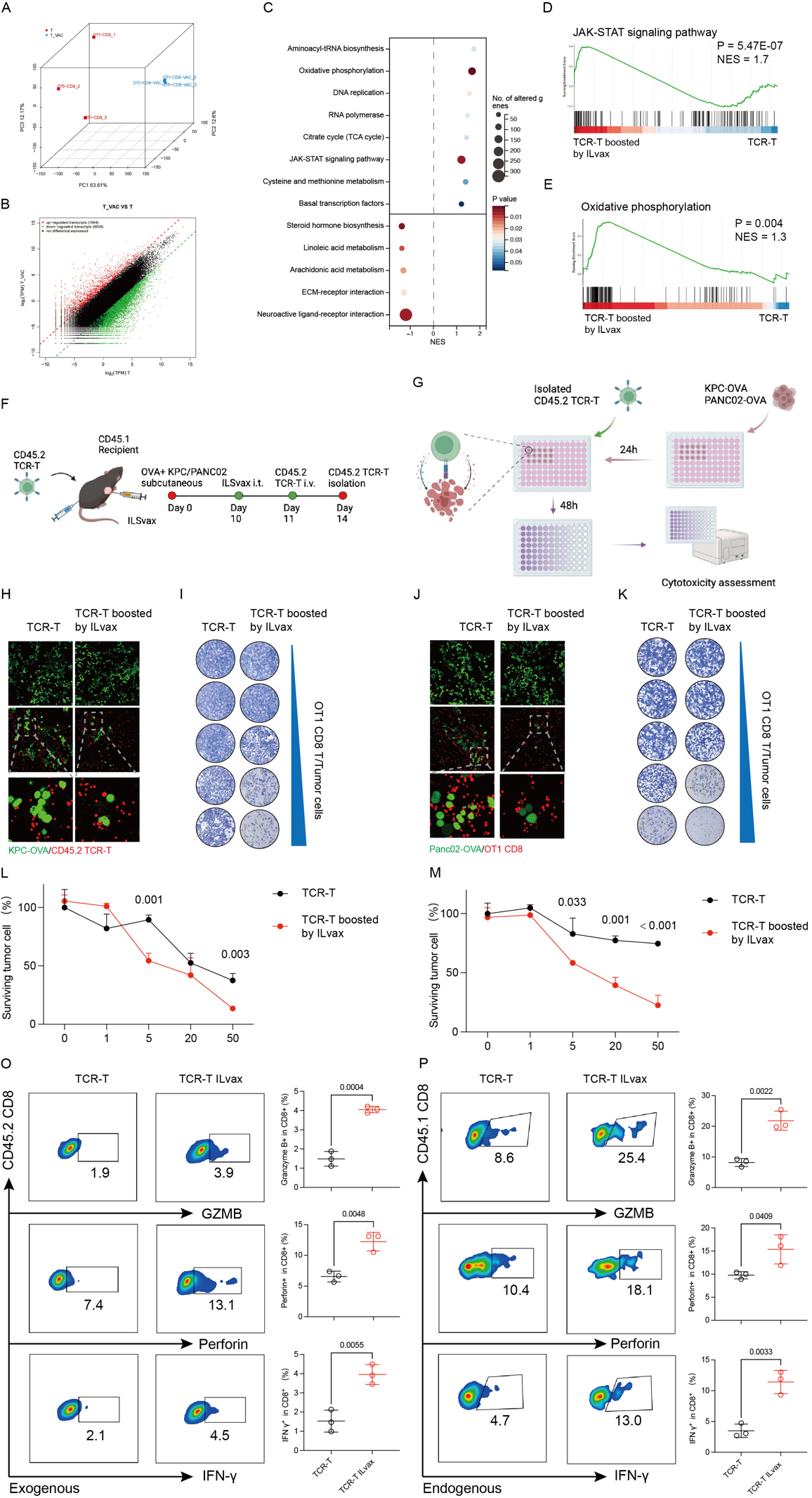


Figure S5. ILvax ablation induces cell-intrinsic enhancements in adoptive T cells functionality (related to Figure 5).

(A) Principal Component Analysis (PCA). (B) Differential expression genes between the PBS- and ILvax- treated tumours. (C, D, and E) GSEA enrichment of DEGs between the PBS- and ILvax- treated tumours. (F) Scheme illustration of ILvax-boosted CD45.2 TCR-T isolation. (G) Scheme illustration of cytotoxicity assessment for TCR-T and ILvax-boosted TCR-T. (H and J) Confocal imaging of KPC-OVA and PANC02-OVA cultured with TCR-T or ILvax-boosted TCR-T. (I and K) Crystal Violet Staining of KPC-OVA and PANC02-OVA treated with TCR-T or ILvax-boosted TCR-T. (L and M) CCK8 analysis of KPC-OVA and PANC02-OVA treated with TCR-T or ILvax-boosted TCR-T, n=3. (O) Representative flow cytometry plot and quantitative analysis of granzyme B, Perforin, and IFN-γ in exogenous CD45.2 TCR-T cells. (P) Representative flow cytometry plot and quantitative analysis of granzyme B, Perforin, and IFN-γ in endogenous CD45.1 CD8^+^ T cells. Data are presented as mean ± SD and p values were determined by two-tailed unpaired student’s t-test (L, M, O, and P). Source data are provided as a Source Data file.

Table S1. Detailed information of antibodies for flow cytometry

| CD11B CD11b Monoclonal Antibody  (M1/70), Brilliant Ultra Violet 395 | eBioscience | Cat# 363-0112-82 |
| --- | --- | --- |
| CD11C CD11c Monoclonal Antibody  (N418), Brilliant Ultra Violet 737 | eBioscience | Cat# 367-0114-82 |
| CD19 Monoclonal Antibody (eBio1D3 (1D3)), Brilliant Ultra Violet 563 | eBioscience | Cat# 365-0193-82 |
| CD4 Monoclonal Antibody (RM4-5), Brilliant Ultra Violet™ 615, eBioscience™ | eBioscience | Cat# 366-0042-82 |
| CD8a Monoclonal Antibody (53-6.7), Brilliant Ultra Violet 805 | eBioscience | Cat# 368-0081-82 |
| BD OptiBuild™ RB780 Rat Anti-Mouse CD3 | BD Biosciences | Cat#755792 |
| Brilliant Violet 785™ anti-mouse CD45 Antibody | Biolegend | Cat#103149 |
| F480 PE/Cyanine7 anti-mouse F4/80 Antibody | Biolegend | Cat#123114 |
| PE anti-mouse CD11c Antibody | Biolegend | Cat#117308 |
| Brilliant Violet 421™ anti-mouse I-A/I-E Antibody | Biolegend | Cat#107632 |
| Spark NIR™ 685 anti-mouse NK-1.1 Antibody | Biolegend | Cat#156530 |
| FITC anti-mouse CD3 Antibody | Biolegend | Cat#100204 |
| PE/Cyanine7 anti-mouse CD8a Antibody | Biolegend | Cat#100722 |
| FITC anti-mouse Ly-6G Antibody | Biolegend | Cat#127606 |
| Brilliant Violet 650™ anti-mouse Ly-6C Antibody | Biolegend | Cat#128049 |
| Brilliant Violet 605™ anti-mouse CD279 (PD-1) Antibody | Biolegend | Cat#135220 |
| PE/Fire™ 640 anti-mouse CD366 (Tim-3) Antibody | Biolegend | Cat#119750 |
| PerCP/Cyanine5.5 anti-mouse CD103 Antibody | Biolegend | Cat#121416 |
| Brilliant Violet 510™ anti-mouse/human CD44 Antibody | Biolegend | Cat#103044 |
| PE anti-mouse CD62L Antibody | Biolegend | Cat#161204 |
| APC anti-mouse H-2Kb bound to SIINFEKL Antibody | Biolegend | Cat#141606 |
| Flex-T™ Biotin H-2 K(b) OVA Monomer (SIINFEKL) | Biolegend | Cat#280051 |
| PerCP/Cyanine5.5 anti-human/mouse Granzyme B Recombinant Antibody | Biolegend | Cat#372212 |
| APC anti-mouse IFN-γ Antibody | Biolegend | Cat#505810 |
| PE anti-mouse Perforin Antibody | Biolegend | Cat#154306 |
| Brilliant Violet 605™ anti-mouse Ki-67 Antibody | Biolegend | Cat#652413 |
| Brilliant Violet 785™ anti-mouse CD69 Antibody | Biolegend | Cat#104543 |
| PE anti-mouse CD197 (CCR7) Antibody | Biolegend | Cat#120106 |

Table S2. Detailed information of antibodies for western blotting

| Jak1 (6G4) Rabbit mAb | Cell Signaling  Technology | Cat#3344 |
| --- | --- | --- |
| Phospho-Jak1(Tyr1034/1035) (D7N4Z) Rabbit mAb | Cell Signaling  Technology | Cat#74219 |
| Stat5 (D2O6Y) Rabbit mAb | Cell Signaling  Technology | Cat#94205 |
| Pstat5 Phospho-Stat5 (Tyr694) (C11C5) Rabbit mAb | Cell Signaling  Technology | Cat#9359 |
| Anti-PGC1 alpha | Abcam | Cat#ab313559 |
| GAPDH Mouse Monoclonal Antibody | Beyotime | Cat# AF0006 |
| HRP goat anti-rabbit IgG | Beyotime | Cat# A0208 |
| HRP Goat anti-mouse IgG | Beyotime | Cat# A0216 |

Table S3. Detailed information of antibodies for IF and IHC

| CD45.2 Purified anti-mouse CD45.2 Antibody | Biolegend | Cat#109802 |
| --- | --- | --- |
| Purified anti-mouse CD370 (CLEC9A, DNGR1) Antibody | Biolegend | Cat#143502 |
| Cd8a CD8α (D4W2Z) XP® Rabbit mAb | Cell Signaling  Technology | Cat#98941 |
| Cd11c CD11c (D1V9Y) Rabbit mAb | Cell Signaling  Technology | Cat#97585 |
| Panck Pan-Keratin (C11) Mouse mAb | Cell Signaling  Technology | Cat#4545 |
| F480 F4/80 (D4C8V) XP® Rabbit mAb | Cell Signaling  Technology | Cat#30325 |
| CD4 (D7D2Z) Rabbit mAb | Cell Signaling  Technology | Cat#25229 |
| Ly-6G (E6Z1T) Rabbit mAb | Cell Signaling  Technology | Cat#87048 |
| Ki-67 (D3B5) Rabbit mAb | Cell Signaling  Technology | Cat#12202 |
| Granzyme B (D6E9W) Rabbit mAb | Cell Signaling  Technology | Cat#46890 |

Table S4. Detailed information of antibody drugs for immune cells depletion

| InVivoMAb anti-mouse CD8α | BioXCell | Cat#BE0004 |
| --- | --- | --- |
| InVivoMAb anti-mouse CD4 | BioXCell | Cat#BE0003-1 |
| InVivoMAb anti-mouse NK1.1 | BioXCell | Cat#BE0036 |
| InVivoMAb anti-mouse PD-1 (CD279) | BioXCell | Cat# BE0273 |
| InVivoMAb anti-mouse CLEC9A (CD370) | BioXCell | Cat#BE0305 |
| InVivoMAb anti-mouse F4/80 | BioXCell | Cat#BE0206 |
| InVivoMab lgG2a isotype | BioXCell | Cat#BE0089 |
| InVivoMab lgG2b isotype | BioXCell | Cat#BE0090 |

Table S5. Detailed information of reagents used in this study

| EasySep™ Mouse Naïve CD8+ T Cell Isolation Kit | STEMCELL | Cat#19858 |
| --- | --- | --- |
| Mito-Tracker Green | Thermo Fisher  Scientific | Cat# M46750 |
| CFSE Cell Division Tracker Kit | BioLegend | Cat#423801 |
| Mouse IFN-γ Precoated ELISPOT Kit | Dakewe | Cat#2210005 |
| D- (+) -Glucose | Sigma-Aldrich | Cat#G8270 |
| L-Glutamine (100X) | Beyotime | Cat#C0212 |
| FCCP | MCE | Cat#HY-100410 |
| Rotenone | MCE | Cat#HY-B1756 |
| Antimycin A | Cayman | Cat#19433 |
| Oligomycin Complex | Cayman | Cat#11341 |
| APC Streptavidin | Biolegend | Cat#405207 |
| PE Streptavidin | Biolegend | Cat#405213 |
| Percoll solution | GE healthcare | Cat# 17-0891-01 |
| Protease inhibitor cocktail | Selleck | Cat# B14001 |
| Phosphatase Inhibitor Cocktail | Selleck | Cat# B15001 |
| Collagenase IV | Thermo Fisher  Scientific | Cat# 17104019 |
| DNase | Sigma-Aldrich | Cat# D5025 |
| Matrigel | Corning | Cat# 356231 |
| D-Luciferin, Potassium Salt | Goldbio | Cat#115144-35-9 |
| Leukocyte activation cocktail | BD biosciences | Cat#550583 |
| BD Horizon™ Fixable Viability Stain 450 | BD biosciences | Cat#562247 |
| BD Horizon™ Fixable Viability Stain 780 | BD biosciences | Cat#565388 |
| DAB chromogen kit | Biocare | Cat#BDB2004 |
| Anti-mouse IgG (H+L), F(ab')2 Fragment (Alexa Fluor® 488 Conjugate) | Cell Signaling  Technology | Cat#4408 |
| Anti-rabbit IgG (H+L), F(ab')2 Fragment (Alexa Fluor® 555 Conjugate) | Cell Signaling  Technology | Cat#4413 |
| Anti-rabbit IgG (H+L), F(ab')2 Fragment (Alexa Fluor® 647 Conjugate) | Cell Signaling  Technology | Cat#4414 |
